# Machine learning-based identification of key biotic and abiotic drivers of mineral weathering rate in a complex enhanced weathering experiment

**DOI:** 10.12688/openreseurope.19252.3

**Published:** 2025-09-11

**Authors:** Iris Janssens, Thomas Servotte, Tullia Calogiuri, Steven Mortier, Harun Niron, Thomas Corbett, Reinaldy P. Poetra, Lukas Rieder, Michiel Van Tendeloo, Abhijeet Singh, Steven Latré, Siegfried E. Vlaminck, Jens Hartmann, Jan Willem van Groenigen, Anna Neubeck, Alix Vidal, Ivan A. Janssens, Mathilde Hagens, Sara Vicca, Tim Verdonck

**Affiliations:** 1 Department of Computer Science, University of Antwerp - imec - IDLab, Antwerp, Belgium; 2 Department of Mathematics, University of Antwerp - imec - IDLab, Antwerp, Belgium; 3 Soil Biology Group, Wageningen University & Research, Wageningen, The Netherlands; 4 Department of Bioscience Engineering, Biobased Sustainability Engineering (SUSTAIN), University of Antwerp, Antwerp, Belgium; 5 Department of Earth Sciences, Uppsala University, Uppsala, Sweden; 6 Center for Earth System Research and Sustainability, University of Hamburg, Institute for Geology, Hamburg, Germany; 7 Faculty of Agriculture, Allied Sciences and Technology, Ganpat University, Mehsana, India; 8 Department of Biology, University of Antwerp, Antwerp, Belgium; 9 Department of Soil Chemistry, Wageningen University & Research, Wageningen, The Netherlands

**Keywords:** enhanced mineral weathering, carbon sequestration, machine learning, explainable AI

## Abstract

**Background:**

The optimization of enhanced mineral weathering as a carbon dioxide removal technology requires a comprehensive understanding of what drives mineral weathering. These drivers can be abiotic and biotic and can interact with each other. Therefore, in this study, an extensive 8-week column experiment was set up to investigate 30 potential drivers of mineral weathering simultaneously.

**Methods:**

The setup included various combinations of rock types and surface areas, irrigation settings, biochar and organic amendments, along with various biota and biotic products such as earthworms, fungi, bacteria and enzymes; each varying in type or species and quantity. The resulting changes in dissolved, solid, and total inorganic carbon (∆TIC), and total alkalinity were calculated as indicators of carbon dioxide removal through mineral weathering. Three machine learning models, Least Absolute Shrinkage and Selection Operator (LASSO), Random Forest and eXtreme Gradient Boosting (XGB) regression, were used to predict these indicators. Dominant drivers of the best performing model were investigated using SHapley Additive exPlanations (SHAP).

**Results:**

SHAP analysis revealed that each CDR indicator was influenced by different factors. However, key drivers were consistently abiotic, though biota also made a significant contribution to the predictions. The most representative CDR indicator, ∆TIC, was predominantly driven by steel slag addition and mixed rock grain sizes but was also substantially impacted by earthworms and microbes.

**Conclusions:**

These findings provide valuable insights into the complex interplay of numerous abiotic and biotic factors that affect mineral weathering, highlighting the potential of machine learning to unravel complex relationships in biogeochemical systems.

## Plain language summary

**The need for carbon capture:**

The increasing concentration of carbon dioxide (CO _2_) in the atmosphere, driven by human activities, is the primary cause of global warming. To limit global temperature rise to below 2°C, CO _2_ emissions need to decline to net zero, and excess CO _2_ must be removed using carbon dioxide removal (CDR) technologies. Many existing carbon capture technologies are energy-intensive and compete for resources such as land, making them challenging to scale. As a result, alternative methods like enhanced weathering are gaining attention due to their potential for CO _2_ removal without these drawbacks.

**Enhanced weathering:**

Natural mineral weathering is a process where carbonic acid, formed from CO _2_ and water, reacts with minerals, breaking them down and capturing carbon in the form of dissolved bicarbonate ions or carbonate minerals. This natural process slowly reduces atmospheric CO _2_ over long time scales. The process can be enhanced, amongst others by grinding minerals to increase their surface area, which leads to an increase in CO _2_ removal, but potentially also yields co-benefits, such as improving soil health. Besides grinding minerals, biota could possibly further enhance weathering, for example by producing organic acids, enzymes and siderophores, or by physically mixing soil and rocks. However, the impact of these biota on mineral weathering and the interactions between multiple drivers of mineral weathering are complex and not yet fully understood.

**Experiment:**

To explore these drivers, an extensive column experiment was conducted with 200 columns per round, running across 10 rounds. Each column contained unique combinations of minerals, organic amendments, and biota, including bacteria, fungi and earthworms, and was irrigated with different flow rates and frequencies. The impact on CDR was assessed using indicators such as changes in dissolved inorganic carbon, solid inorganic carbon, total inorganic carbon, and total alkalinity.

**Machine learning analysis:**

Given the complexity of the dataset, machine learning (ML) was used to predict the CDR indicators. To address the "black box" nature of the used ML models, they were combined with explainability methods, which identified the most influential drivers of mineral weathering. These drivers were mainly abiotic, but several biota also had a substantial impact on the predictions.

This study demonstrates how combining large-scale experiments with ML and explainability techniques can provide valuable insights into optimizing enhanced weathering strategies for CDR.

## Introduction

The increasing concentration of carbon dioxide (CO _2_) in the atmosphere due to human activities is the main driver of climate warming ( IPCC, 2018). To limit global warming to below 2°C, CO _2_ emissions must decline to net zero as quickly as possible and excess CO _2 _must be removed from the atmosphere using carbon dioxide removal (CDR) technologies ( IPCC, 2018). In response, scientists across various fields are exploring and optimizing CDR technologies. One such CDR technology is enhanced weathering (EW), which accelerates the naturally occurring process of mineral weathering ( Hartmann *et al.*, 2013; Köhler *et al.*, 2010). In mineral weathering, dissolved carbonic acid (which is in equilibrium with gaseous CO _2_) reacts with minerals to form water-soluble bicarbonate ions, increasing dissolved inorganic carbon (DIC), part of which may not be degassed back to the atmosphere for millennia ( Berner, 2004; Hartmann *et al.*, 2013; Köhler *et al.*, 2010). During the mineral weathering process, disintegrating rocks also release cations and silica ( Beerling *et al.*, 2018; White & Buss, 2014), and part of the formed DIC and cations (e.g., Ca ^2+^) may react and precipitate carbonate minerals, forming solid inorganic carbon (SIC) and releasing part of the CO _2_ originally sequestered ( Hartmann *et al.*, 2013).

Natural weathering rates can be vastly accelerated by, amongst others, grinding minerals to increase reactive surface areas, which gave rise to the term EW. EW could exploit significant quantities of waste rock streams ( Renforth, 2019) and may have agricultural co-benefits ( Beerling *et al.*, 2018). Moreover, EW does not compete for land with other CDR technologies such as soil organic carbon sequestration, reforestation or bio-energy production with carbon capture and storage (BECCS) and may thus be combined with these CDR technologies ( Horton *et al.*, 2021; Janssens *et al.*, 2022). However, care must always be taken to ensure that the added minerals will not increase heavy metal availabilities beyond risk-bearing thresholds ( Dupla *et al.*, 2023; Gastmans *et al.*, 2016; Vienne *et al.*, 2022).

To date, efforts to accelerate mineral weathering have predominantly focused on a suite of abiotic variables, such as mineral type and grain size. The potential impacts of biotic factors are often overlooked ( Vicca *et al.*, 2022), despite the fact that studies have shown that biota can potentially boost weathering. For example, fungal and bacterial species can actively facilitate the weathering of silicate minerals ( Berner, 2004; Finlay *et al.*, 2020). One mechanism by which biota accelerate mineral weathering is their respiration, increasing the CO _2 _pressure of the system. Consequently, due to Henry’s law, concentrations of the weathering agent carbonic acid increase and CDR is stimulated ( Dontsova *et al.*, 2020; Gerrits *et al.*, 2020; Perez-Fodich & Derry, 2019; Vicca *et al.*, 2022). Besides CO _2_ pressure, a manifold of biotic factors, such as enzymes and siderophores (chelators) could further stimulate weathering ( Vicca *et al.*, 2022; Wild *et al.*, 2022). To date, the complexity of biota-weathering relationships is far from resolved ( Milici *et al.*, 2024), and Deng *et al.* (2023) recommended future research aimed at improving understanding of the roles of biotic-abiotic interactions in controlling EW efficiency.

In soils, quantification of EW is exceptionally difficult, among others because the increases in DIC and total alkalinity (TA) following mineral weathering can be undone by secondary mineral precipitation, which may remove DIC and TA from the soil solution ( Amann *et al.*, 2022; Kelland *et al.*, 2020; Niron *et al.*, 2024). In real-world situations, e.g., in agricultural experiments, this is aggravated by the fact that soils are extremely heterogeneous in almost every aspect, often rendering soil samples poorly representative for the total soil. For this reason, EW experiments are often conducted in controlled environments, such as column experiments, to ease the quantification by homogenizing many of the variables potentially affecting EW rates. Given the myriad of abiotic and biotic drivers at play, along with their broad potential ranges, it is virtually impossible to test every conceivable combination of experimental settings in a repeated multifactorial experiment. Therefore, experiments that aim to simultaneously test the many possible drivers and their interactions inevitably result in complex datasets that make traditional statistical methods ineffective. The mostly unreplicated driver combinations in such complex experiments, as well as relatively small amounts of data compared to the number and possible ranges of possible drivers, open the door to a machine learning (ML) approach.

Most EW studies focused on the effects of only one driver or at best a combination of two or three drivers. This leaves the interaction effects of multiple drivers on EW largely unknown. Here, to simultaneously test the impacts of 30 potential biotic and abiotic drivers of EW, each with multiple different added amounts or size classes, an extensive column experiment was set up with unique combinations of rock powders, organic amendments, biota and biotic products ( Calogiuri *et al.*, 2023). These column inputs were chosen to explore several research questions. For example, steel slags are known to weather faster than basalt, but may lead to unlivable conditions for biota due to potential rapid increases in pH and heavy metals. Therefore, various rock types were assessed individually and in mixtures. Second, studies have shown that finer grain sizes increase weathering rates due to increased reactive surface area ( Basak *et al.*, 2021; Vanderkloot & Ryan, 2023). However, fine grain sizes may block or retard water infiltration, leading to pore water oversaturation and precipitation of newly formed minerals that may even inhibit further weathering ( Amann *et al.*, 2022). In our experiment, grain sizes were therefore varied and often mixed. Additionally, as biochar may scavenge base cations from solution and theoretically shift the equilibrium of mineral weathering ( Amann & Hartmann, 2019), biochar was added to some of the applied mixtures. To test the impact of biota on the weathering rate, species of bacteria and fungi that were previously shown to induce mineral weathering, amongst others through production of enzymes and siderophores and through formation of biofilms on rock surfaces ( Corbett *et al.*, 2024; Gerrits *et al.*, 2020; Gostincar *et al.*, 2014; He *et al.*, 2021; Kim *et al.*, 2005; Niron *et al.*, 2024; Nwoko *et al.*, 2021), were added to the mixtures. Also earthworms were added, primarily for their soil mixing activity, removing precipitated secondary minerals from the silicate surfaces, and to stimulate microbial activity ( Vicca *et al.*, 2022). To support the activity of the microbes and earthworms and fuel their respiration, different types of organic amendments were added. Moreover, we tested the effect of adding nitrogen and phosphorus on EW via a potential effect on microbial activity. Last, the addition of enzymes and organic acids was assessed. The columns were then irrigated at different frequencies and throughput rates. Due to the open column setup, CO _2_ and thus CDR could not be directly measured. Instead, the impact of the inputs on CDR through mineral weathering was assessed based on measurements of SIC, DIC and TA, and on the inorganic carbon (IC) and TA initially present in the system. These are commonly used indicators of silicate weathering ( Clarkson *et al.*, 2024). The change in DIC leaching and in SIC sum up to the total inorganic carbon (TIC) sequestration. TA can provide further insight into differences in weathering dynamics between treatments.

The resulting data was thereafter analysed using an ML approach that combined predictive modeling and model explainability, as has previously been done for other high-dimensional setups, e.g., in pharma industry ( Bannigan *et al.*, 2023; Luo *et al.*, 2024), health industry ( Raihan *et al.*, 2023) and to study water- and air quality ( Bacanin *et al.*, 2024; Park *et al.*, 2022; Wang *et al.*, 2021). We selected three predictive ML models; least absolute shrinkage and selection operator regularization (Lasso), Random Forest (RF), and eXtreme Gradient Boosting (XGB), which are popular tools in literature and which we chose for the following desirable characteristics; Lasso performs linear regression combined with feature selection by penalizing less important variables, which is particularly useful in high-dimensional settings where many predictors may be irrelevant ( Santosa & Symes, 1986; Tibshirani, 1996). This feature selection is achieved through L1 regularization, i.e., by adding a penalization term to the L1-norm of the coefficients in the regression model. This results in a sparse solution where some coefficients are exactly zero ( Tibshirani, 1996). Consequently, Lasso excludes unimportant predictors, which simplifies the model and enhances the accuracy. Weathering reactions, such as those promoted in enhanced weathering experiments, are driven by thermodynamics and are, in absence of biotic interactions, highly linear such that their kinetics can be modelled well ( Curtis, 1976; Heřmanská *et al.*, 2022; Heřmanská *et al.*, 2023). Model outputs may be transformed to yield linear relationships or normal distributions, enabling parametric statistical approaches to elucidate results from experiments or variability in time series. However, *in situ*, microbes will respond to the altered environmental conditions and availability of elements in soils, among others by altering their respiration, exudation of organic acids, or taking up reaction products. These microbial responses thus elicit a suite of feedbacks that the current generation of geochemical models cannot reproduce, rendering some geochemical reactions unpredictable and highly non-linear. Under these real-world conditions where reaction kinetics cannot be reproduced by existing models, machine learning that can cope with non-linear relationships may enable the attribution of observed changes in weathering reactions to potential drivers.

RF and XGB allow modeling nonlinear relations and employ ensemble tree learning to improve the prediction accuracy ( Chen & Guestrin, 2016; Ho, 1995). RF achieves this through bootstrapping decision trees and the final prediction is obtained by averaging the outputs of all the trees, effectively reducing overfitting and increasing model robustness ( Ho, 1995). As a result, RF is increasingly used for ecological applications, e.g., Xie *et al.* (2021); Shi *et al.* (2021). XGB is an advanced boosting technique that refines its predictions by iteratively training new trees to correct the errors of the previous trees, often leading to superior performance ( Chen & Guestrin, 2016). Furthermore, XGB includes mechanisms for regularization, which helps prevent overfitting by controlling the complexity of the model, making it both a flexible and robust choice for predictive modeling in ecological and environmental studies. Although XGB often outperforms RF, application of XGB in ecology (e.g., Huang *et al.* (2019); Park *et al.* (2022); Valavi *et al.* (2022)) is less prevalent. Other ML models have shown little added value compared to XGB on small, tabular datasets; with e.g., neural networks underperforming on such datasets and proving harder to explain ( Shwartz-Ziv & Armon, 2022), and support vector machines often being more complicated to tune and not as accurate.

The main drawback of RF and XGB is that they, like most ML models, are black box models, meaning that they are not inherently interpretable ( Lucas, 2020). This drawback can be largely overcome by combining the black box ML models with explainability techniques such as Shapley Additive exPlanations (SHAP) ( Lundberg *et al.*, 2017) or Local Interpretable Model-agnostic Explanations (LIME) ( Ribeiro *et al.*, 2016). These techniques provide information on the importance of the input features to the predictions and have previously been used in ecology to study, amongst others, species distribution models ( Ryo *et al.*, 2021), water ecosystems ( Kruk *et al.*, 2021), water quality ( Park *et al.*, 2022; Wang *et al.*, 2021), tree species richness ( Brugere *et al.*, 2023) and plant phenology ( Mortier *et al.*, 2024). We used SHAP values to enhance the interpretability of the black-box models, as they are model-agnostic, can account for interactions between features and are often used in ecology ( Brugere *et al.*, 2023; Kruk *et al.*, 2021; Mortier *et al.*, 2024).

Notwithstanding that DIC resembles TA in most organic-poor systems, in organic-rich systems, TA has an inorganic and organic component ( Song *et al.*, 2020). Moreover, in a purely abiotic system, DIC and SIC are in equilibrium (yet controlled by kinetic rate constants of carbonate precipitation and dissolution), implying that a driver that accelerates or decelerates SIC formation would induce opposite changes in DIC and SIC, relative to comparable columns without the driver. However, biota can influence these kinetic rates, e.g., by exuding acids or the enzyme carbonic anhydrase, or by kickstarting precipitation due to cells functioning as carbonate nucleation sites. Therefore, we hypothesise that the prediction of change in amount of SIC (∆SIC), change in amount of DIC (∆DIC) and change in amount of TA (∆TA) will be driven by different features.

This study aims to uncover how mineral weathering results in CDR by combining insights in the key drivers of four different mineral weathering indicators. First, this paper summarizes the extensive column experiment designed to test the effects of 30 potential abiotic and biotic drivers on EW. Next, the weathering process is evaluated by calculating four different indicators of mineral weathering rate, i.e., ∆DIC, ∆SIC, change in amount of TIC (∆TIC) and ∆TA. To address the challenges posed by the complexity and dimensionality of the dataset, we first employed a range of regression models, including Lasso, RF, and XGB, to predict the CDR indicators based on the input materials of the columns. Finally, by integrating these ML models with explainability through SHAP values, we gained deeper insights into the underlying mechanisms of mineral weathering, making this approach a powerful tool for optimizing enhanced weathering strategies.

## Methods

### Column experiments

In order to elucidate the influence of abiotic and biotic factors on EW, ten rounds of column experiments were conducted in a climate chamber set at 25°C and 30% relative humidity. Each round consisted of 200 columns, each of which comprised a cylinder (7 cm diameter x 15 cm height) filled with a mixture of a wide range of combinations of abiotic and biotic inputs. Full details of the experimental setup are provided in Calogiuri *et al.* (2023). In summary, each mixture contained 360–400g of rock powder, consisting of 1-3 different rock types with different possible grain sizes (Table A11 in Extended Data ( Janssens *et al.*, 2025)). Here, the rock types were a peridotite rock with about 90% olivine content, a steel slag, and two rocks in the field of basalt and basanite/tephrite according to the TAS diagram (see Figure A1 in Extended Data). The rocks and slags used are detailed in Tables A3-A7 in Extended Data.

To this mixture, different amounts of biochar, organic amendments, living or dead earthworms, bacteria, fungi, enzymes, and additional N (in the form of urea or NH _4_Cl) and P (in the form of KH _2_PO _4_) could be added. The biochar was produced from natural wood (Verora, Germany; see Table A8 for its properties). The organic amendments were wheat straw (Pets Place, the Netherlands), co-digestate of pig manure and biowaste (Groene Mineralen Centrale, the Netherlands) or a mixture dominated by alfalfa with additions of wheat and grape molasse (hereinafter referred to as ’alfalfa mixture’ for simplicity; Pets Place, the Netherlands). The elemental compositions of the biochar and organic amendment are listed in Table A9 in Extended Data.

The earthworm species were Allolobophora chlorotica (A. chlorotica) and/or Aporrectodea caliginosa (A. caliginosa)), and were collected from the park De Blauwe Bergen in Wageningen, the Netherlands (51°58'51.8"N 5°39'38.0"E) at the start of each round of experiments. To prevent the earthworms from escaping, a mesh was placed on top of the columns. The bacteria were *Bacillus subtilis str.* Marburg (DSM 10 ^T^) ( *B. subtilis*) and/or Cupriavidus metallidurans str. CH34 (DSM 2839 ^T^) ( *C. metallidurans*), both sourced from Leibniz Institute DSMZ, Germany. The fungi were *Knufia petricola str.* A95 (Wollenzien & de Hoog) Gorbushina and Gueidan 2013 (CBS 123872) ( *K. petricola*), sourced from Westerdijk Institute, the Netherlands, *Aureobasidium pullulans str.* 316 (de Bary & Löwenthal) Arnaud (DSM 3497) ( *A. pullulans*) and/or *Suillus variegatus* (Sw.) Kuntze (DSM 1752) ( *S. variegatus*), both sourced from Leibniz Institute DSMZ, Germany. Last, the enzymes were laccase, urease and/or carbonic anhydrase.

In some of the treatments, the microbes were premixed with the organo-mineral mixture; inoculation was done at the start only or also after four weeks; and in some treatments autoclaving was performed to kill the any/all pre-existing microbes in the rocks and organic amendments (126–135°C for 4 hours) to maximize the colonization potential of the added microbial species. For eight weeks, natural ground water was applied via a downflow irrigation system, with different water throughput rates and frequencies (see Tables S1-S2 in Extended Data for its properties). A complete overview of the inputs and their added amounts is listed in Table 1. To collect the leachates, each column was placed above a jerrycan cooled at 4°C to minimize microbial growth and dissolved organic carbon consumption ( Kirschbaum, 1995).

**Table 1.** Model features with their added amounts.

Model features	Description	Amounts
**Irrigation**		
irrigation flow rate	Irrigation flow rate	50, 100, 150 ml day ^-1^
irrigation frequency	Irrigation frequency	1, 2, 5 times day ^-1^
**Rocks**		
basalt SA	Total surface area of basalt rocks (see Extended data - Eq. B.18 ( Janssens *et al*., 2025))	∈ [0,4231] *m* ^2^
basanite/tephrite SA	Total surface area of basanite/tephrite rocks (see Extended data - Eq. B.18 ( Janssens *et al*., 2025))	∈ [0,759] *m* ^2^
peridotite SA	Total surface area of peridotite rocks (see Extended data - Eq. B.18 ( Janssens *et al*., 2025))	∈ [0,2820] *m* ^2^
steel slag SA	Total surface area of steel slags (see Extended data - Eq. B.18 ( Janssens *et al*., 2025))	∈ [0,5299] *m* ^2^
mixed grain size	Whether multiple grain sizes were mixed or not	true/false
rock+biochar mass	Total mass of the rocks and biochar addition	400, 440 g
**Biochar**		
biochar mass	Mass of biochar from natural wood	0, 4, 10, 20, 40 *g*
**Organic amendments**		
straw mass	Addition of wheat straw	0, 10 g
digestate mass	Addition of co-digestate of pig manure and biowaste	0, 10 g
alfalfa mixture mass	Addition of a mixture dominated by alfalfa with additions of wheat and grape molasse	0, 10 g
**Earthworms**		
# earthworms	Number of initially live earthworms	∈ [0, 12]
*chlorotica* ratio	Ratio of initially live *Allolobophora chlorotica* earthworms w.r.t. total number of initially live earthworms (see Extended data - Eq. B.19 ( Janssens *et al*., 2025))	∈ [0,1]
# † earthworms	Number of initially dead earthworms	∈ [0, 12]
† *chlorotica* ratio	Ratio of initially dead *Allolobophora chlorotica* earthworms w.r.t. total number of initially dead earthworms (see Extended data - Eq. B.20 ( Janssens *et al*., 2025))	∈ [0, 1]
**Fungi**		
# *K. petricola*	Inoculum density of *Knufia petricola str.* A95 (Wollenzien & de Hoog) Gorbushina and Gueidan 2013 (CBS 123872)	∈ [0,5.4 *e*8] cells
# *A. pullulans*	Inoculum density of *Aureobasidium pullulans str. 316 (de Bary & Löwenthal) Arnaud (DSM 3497)*	∈ [0,5 *.*4 *e*8] cells
# *S. variegatus*	Inoculum density of *Suillus variegatus (Sw.) Kuntze (DSM 1752)*	∈ [0,4 *.*3 *e*8] cells
**Bacteria**		
# *B. subtilis*	Inoculum density of *Bacillus subtilis str. Marburg (DSM 10T)*	∈ [0,4 *.*7 *e*11] cells
# *C. metallidurans*	Inoculum density of *Cupriavidus metallidurans str*. CH34 (DSM 2839T)	∈ [0,4 *.*0 *e*11] cells
premixed	Whether the microbes were premixed with the organo-mineral mixture	true/false
sterilized	Whether the rocks and organic amendments were autoclaved before adding biota	true/false
halftime inoculation	Whether the bacteria and fungi were added after 4 weeks	true/false
**Enzymes**		
# laccase	Laccase concentration	∈ [0,300] U
# carbonic anhydrase	Carbonic anhydrase concentration	∈ [0,3998] U
# urease	Urease concentration	∈ [0,370] U
**Extra additions**		
# P	Extra phosphorus addition	0, 0.3 mmol
# NH _4_Cl	Extra ammonium chloride addition	0, 0.3 mmol week ^-1^
# urea	Extra urea addition	0, 0.15 mmol week ^-1^

At the end of each round of experiments, samples were taken from both the solid mixture in the column and the leachate, from which several quantities were measured to study the CDR due to mineral weathering. To obtain a representative sample from the solid mixture, the mixture was homogenized and samples were taken using the coning and quartering method ( Campos & Campos, 2017). After performing loss of ignition (LOI) on the solid sample (for three hours at 550°C; Koorneef *et al.*, 2023), its concentration of carbon, i.e., SIC concentration (mass%), was measured (using C analysis, Flash 2000 CN Soil Analyser, Interscience, Antwerp, Belgium). In the leachate sample, the concentrations of TA (µmol l ^-1^) and DIC (mg l ^-1^) were measured (using Automated Gran titration, Titrando (600 series), Metrohm, Hamburg, Germany; and FormacsHT TOC analyser, Skalar, Antwerp, Belgium). The uncertainty of the DIC measurements is given with around 20% and that of TA with 2%.

Since (bi)carbonates and alkalinity were already present in some of the input materials (minerals, organic amendments), their SIC concentration was measured to be able to isolate the IC in the solid mixture resulting from mineral weathering. To assess the amount of DIC and TA resulting from processes other than mineral weathering, such as weathering and DIC release of trace carbonates in organic feedstocks, control experiments were conducted in parallel. In these control experiments, DIC and TA leaching from columns containing only 400g sand, as well as columns filled with 400g sand and each type of organic amendments, was quantified (see Tables A10-A11 in Extended Data). Further details regarding the design and construction protocol of the column experiments can be found in Calogiuri *et al.* (2023).

### Data preprocessing

To examine CDR and the underlying mineral weathering process, we focused on four targets; ∆ *SIC*, ∆ *DIC*, the sum of the two, coined ∆ *TIC*, and ∆ *TA*; all expressed in *mmol* (per column over the 8-week experiment). Their derivations can be found in Extended Data, section B.1. In short, ∆ *SIC* ( *mmol*) was calculated from the measured *SIC* concentration ( *mass*%) at the end of each experiment and the mass of the solid bed ( *g*), and was corrected for the weight loss due to LOI and for the *SIC* that was initially present in the abiotic components and organic amendments. The two targets measured in the leachate, ∆ *DIC* and ∆ *TA* ( *mmol*), were calculated using the irrigated volume ( *l*) and, respectively, the measured *DIC* ( *mg l* ^-1^) and *TA* ( *µmol l* ^-1^) concentrations in the leachate. To obtain the amounts stemming from mineral weathering, the concentrations originating from the initial carbonates in the rocks and organic amendments, which were approximated using the measurements of the control experiments, were subtracted. Last, ∆ *TIC* was obtained by summing ∆ *SIC* and ∆ *DIC*.

Next, the dataset was preprocessed. Columns with missing target data due to measurement errors were omitted, as well as columns where an experimental error occurred, such as flooding or loss of leachates. Experimental treatment combinations that were repeated were compared and outlying repeats were discarded if the standard deviation of the set of repeats was large with respect to the spread of the data (larger than 75% of the interquartile range). In rounds 1 to 3, additional samples for chemical analyses were taken from the leachate after three weeks, which affected the measurements after eight weeks. Therefore, data of rounds 1 to 3 were omitted for the prediction of Δ TA, Δ DIC and Δ TIC. Because the leachate sampling happened in the leachate container, it did not impact the column with solid matter above, whose data were still used. As we realized early in the project that the leachate data from the first three rounds could not be used in this analysis, many input combinations covered in these first three rounds were repeated in later rounds. As a result, this information was not lost for the ∆DIC, ∆TA and ∆TIC models. While the ∆SIC data contained more replicates, these were properly dealt with in the cross validation (CV) approach (see next section, 'Predictive models') to avoid bias in our analyses. Therefore, we believe that excluding the first three rounds did not introduce biases in this study. This resulted in 749 datapoints for ∆ *DIC*, 1202 for ∆ *SIC*, 725 for ∆ *TIC* and 593 for ∆ *TA*. Next, the input features were defined ( Table 1); e.g., the mix of rock powders was translated to features that represented the total surface area (SA) of each rock type (see section B.2 in Extended Data) and whether or not rock powders with different grain sizes were mixed. This resulted in a mix of boolean and numerical features, which were standardized ( Table 1).

### Predictive models

To uncover the optimal conditions for enhanced mineral weathering, ML was used to predict the CDR indicators; ∆DIC, ∆SIC, ∆TIC and ∆TA. To this end, three different regression models, Lasso, RF and XGB, were fitted to the data using Python (version 3.10), and its scikit-learn ( Pedregosa *et al.*, 2011) and XGBoost ( Chen & Guestrin, 2016) libraries.

While ML models are often trained and evaluated using cross validation (CV), doing this to both optimize the model’s hyperparameters and evaluate the model can lead to an optimistically biased estimate of the model performance ( Stone, 1974). Therefore, in this study, the selection of the optimal hyperparameters, model training and performance evaluation were done using a nested 10×10 CV approach. This approach comprised two levels of CV: a 10-fold CV inner loop for hyperparameter tuning and model training, and a 10-fold CV outer loop for validation. First, in the outer loop, the data was split into 10 training and test sets using 10-fold CV. This entailed splitting the data into 10 folds. To avoid instability of the models due to unbalanced splits, the splitting was done using stratified sampling, with the extra constraint that the absolute value of the coefficient of determination ( *R ^2^*) of the naive prediction (i.e., predicting the mean target value), which should be zero for balanced datasets, was smaller than 0.02. This is done in order to avoid that evaluation metrics are too strongly impacted by outlying values (i.e., very large or very small target values). The restriction based on the naive *R ^2^* makes sure that the train and test split have a similar spread in target values. Next, nine folds were grouped together to form the training set, while the remaining fold served as the test set. Subsequently, this training set underwent a second 10-fold CV in the inner CV loop; being further divided into 10 folds using stratified sampling, that were combined into 10 training and validation sets. These inner-loop training and validation sets were then used for hyperparameter tuning using a grid search, wherein the hyperparameters that led to the best mean absolute error (MAE) on the validation set were selected. The grid search iteratively explored all possible combinations of the varied hyperparameters (listed in Table C1 in Extended Data). This resulted in one model with optimal hyperparameters, which was then evaluated using the remaining test set in the outer CV loop, thereby concluding the first iteration of the outer loop. This process was repeated 10 times, each time with another training and test set, resulting in ten trained models and ten sets of performance metrics. Finally, model performance was assessed by averaging the performance metrics of these ten models. Besides the MAE, we evaluated the performance of the models using the *R ^2^* and the mean squared error (MSE).

### SHAP value analysis

The models described above allow predicting the four target variables, but we also aimed to understand how these predictions come about and which are the dominant drivers. One of the models, Lasso, is a white box model that thus gives insight in the drivers, but the other two applied models, XGB and RF, are not inherently interpretable. We therefore applied explainable artificial intelligence (xAI), more specifically SHAP values, to the best performing model. SHAP values quantify the impact of each feature to the prediction for each datapoint as well as the direction of the impact. SHAP values are determined by assessing the effect of including and excluding each feature on the prediction, while taking all other features into account ( Lundberg *et al.*, 2017). The main advantages of this approach are that SHAP values are easy to interpret, account for interactions between features and are model-agnostic ( Lundberg *et al.*, 2017). The SHAP values were calculated using Python’s SHAP package ( Lundberg *et al.*, 2017). This was done for each test set within the outer CV loop, resulting in one set of SHAP values for every datapoint. Adding steel slag seemed to result in very different scales of SIC and DIC. Therefore, SHAP values were also calculated for the subsets with and without steel slag within each test set.

A useful characteristic of SHAP values is that they are additive, meaning that the SHAP values of all features of a certain datapoint sum to the difference between the predicted value of that datapoint and the mean prediction ( Lundberg *et al.*, 2017). We used this additivity to explore the general impact of a group of features (e.g., rocks, organic amendments or biota) on the predictions. For each datapoint i, the SHAP value of a group of features G can be calculated using

$$SHAP_{G}^{i}=\sum_{feature\in G}SHAP_{feature}^{i}\;(2.1)$$

To then determine a general importance of the group of features, we averaged the absolute value of the group’s SHAP value over all n datapoints:

$$SHAP_{G}=\frac{1}{n}\sum_{i}\left|SHAP_{G}^{i}\right|\;(2.2)$$

This group importance does not account for direction of the impact (accelerating or decelerating CDR), but instead represents the general importance of making a good selection of the features within the group on the predictions.

## Results and discussion

### Data

**Figure 1.**
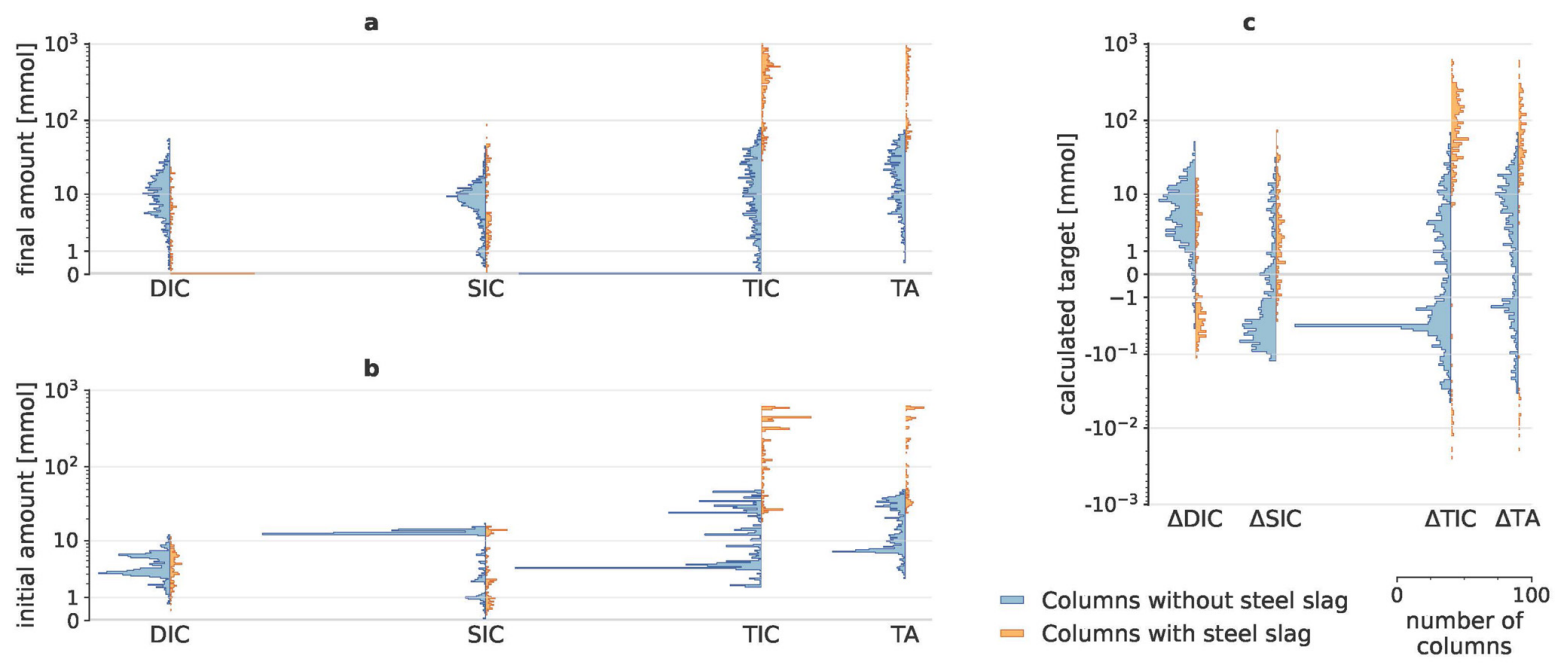
Calculated carbon dioxide removal indicators. Histograms of initial and final dissolved inorganic carbon (DIC), solid inorganic carbon (SIC), total inorganic carbon (TIC) and total alkalinity (TA); with their resulting changes. As adding steel slags resulted in very different scales of SIC, DIC and TA, results for columns with and without steel slags are visualized separately in orange and blue, respectively. ( **a**) shows the amounts that were present in the batches at the end of the experiment; ( **b**) depicts the initial amounts, i.e., the amounts that were added to the system through addition of rocks, organic amendments and throughput water; ( **c**) illustrates the resulting values of the four targets, i.e., the changes in amounts that can be attributed due to mineral weathering.

This study conducted a complex enhanced weathering experiment to reveal the importance of 30 potential drivers in a strongly uneven experimental design with 2000 columns. Initial amounts of DIC, SIC, TIC and TA, added to the system through addition of minerals, biochar, organic amendments and throughput water, could be substantial, especially for SIC ( Figure 1b), which mainly originated from the rocks (Table A11 in Extended Data). After taking these initial amounts into account, the calculated targets (∆SIC, ∆DIC, ∆TIC and ∆TA) only represent the change due to mineral weathering. Negative target values ( Figure 1c) indicate that in some batches, carbon was either converted from one form to another (e.g., SIC to DIC), or carbon precipitated or released to the atmosphere as CO _2_. ∆TIC predominantly consisted of ∆SIC when steel slag was added, though for the columns without steel slag, ∆DIC became more relevant to ∆TIC.

### Model performance

Considering the huge number of possible combinations of column inputs, combinations were generally not repeated to explore the parameter space as much as possible. The resulting uneven experimental design prompted an ML approach to analyse the dataset. To predict the four CDR indicators (∆DIC, ∆SIC, ∆TIC and ∆TA), we selected one linear model, Lasso, because of its simplicity and inherent explainability, and two ensemble models, RF and XGB, for their proven performance in high dimensional non-linear tabular data and their limited data requirements. Both XGB and RF significantly outperformed the naïve prediction, which is a baseline model that uses the mean target value as its prediction, and in many cases also significantly outperformed the Lasso models ( Table 2). For all models, the standard deviations of the performance metrics of the 10 models in the outer CV loop were relatively high ( Table 2), which can be attributed to both the relatively small amount of data - compared to the explored input space - and the noise that was present in the measurements (see section B.3 in Extended Data).

**Table 2.** Performance of the predictions of the carbon dioxide removal indicators. Mean absolute error (MAE), mean squared error (MSE), root mean squared error (RMSE), and determination coefficient (R ^2^) of the prediction of the changes in amount of dissolved inorganic carbon (∆DIC), solid inorganic carbon (∆SIC), total inorganic carbon (∆TIC) and alkalinity (∆TA), using Least Absolute Shrinkage and Selection Operator (Lasso), Random Forest (RF), and eXtreme Gradient Boosting (XGB) regression. The fourth, naive, regressor, predicts the mean target value for the whole training set and is added as a baseline. Depicted values are the mean and standard deviation of the performance metrics of the 10 models of the outer loop of the nested cross-validation. For each target, the model with best average performances is indicated in blue.

Target	Regressor	MAE	MSE	RMSE	R ^2^
ΔDIC [mmol]	Lasso	3.0 ± 0.4	20 ± 5	4.4 ± 0.6	0.62 ± 0.09
	RF	2.9 ± 0.5	19 ± 8	4.3 ± 0.9	0.62 ± 0.17
	XGB	2.4 ± 0.4	16 ± 9	3.9 ± 1.0	0.68 ± 0.20
	Naive	5.4 ± 0.6	54 ± 14	7.3 ± 0.9	0.00 ± 0.01
ΔSIC [mmol]	Lasso	25.9 ± 3.3	2776 ± 1136	51.82 ± 10.04	0.42 ± 0.10
	RF	17.9 ± 3.5	1915 ± 988	42.74 ± 9.93	0.61 ± 0.09
	XGB	17.4 ± 2.5	1752 ± 690	41.16 ± 8.02	0.63 ± 0.12
	Naive	39.3 ± 3.9	4864 ± 1939	68.71 ± 12.58	- 0.01 ± 0.01
ΔTIC [mmol]	Lasso	24 ± 4.4	2571 ± 1793	48.2 ± 16.6	0.32 ± 0.15
	RF	17.4 ± 5.6	1999 ± 1915	40.9 ± 19.1	0.53 ± 0.21
	XGB	15.6 ± 5.1	1563 ± 1810	35.6 ± 18.2	0.62 ± 0.20
	Naive	29.6 ± 3.9	3690 ± 2323	58.4 ± 17.6	-0.01 ± 0.01
ΔTA [mmol]	Lasso	8.0 ± 1.0	243 ± 100	15.3 ± 3.3	0.41 ± 0.28
	RF	5.7 ± 1.1	125 ± 54	11.0 ± 2.3	0.67 ± 0.23
	XGB	5.3 ± 1.2	119 ± 54	10.7 ± 2.5	0.70 ± 0.17
	Naive	11.5 ± 1.5	471 ± 230	21.1 ± 5.6	-0.01 ± 0.01

Lasso is a white-box model and therefore can be very useful. For the prediction of ∆DIC for instance, Lasso had a performance close to that of the other two models ( Table 2). The resulting regression coefficients yield valuable, directly interpretable information. For example, according to the Lasso model, the surface area of added peridotite had the largest effect on ∆DIC, followed by amendment of the alfalfa mixture and straw, and a suite of abiotic and biotic drivers (see Table C2 in Extended Data). However, for the other targets, Lasso exhibited poor performances, with average *R ^2^* values below 0.5 ( Table 2). Lasso is limited to capturing linear relationships, and while linear regression is a popular tool in ecology, biochemical processes are typically nonlinear ( Levin, 1998; Manzoni & Porporato, 2007). RF and XGB allow modeling nonlinear relations and employ ensemble tree learning to improve the prediction accuracy. While RF achieves this through bootstrapping decision trees, XGB iteratively trains new trees to correct the errors of the previous trees, often leading to superior performance. Indeed, although the difference with RF was often small, XGB achieved the best average performance metrics ( Table 2). Therefore, the remainder of our analysis is conducted using XGB.

### Drivers of enhanced weathering using Shapley Additive exPlanations

**Figure 2.**
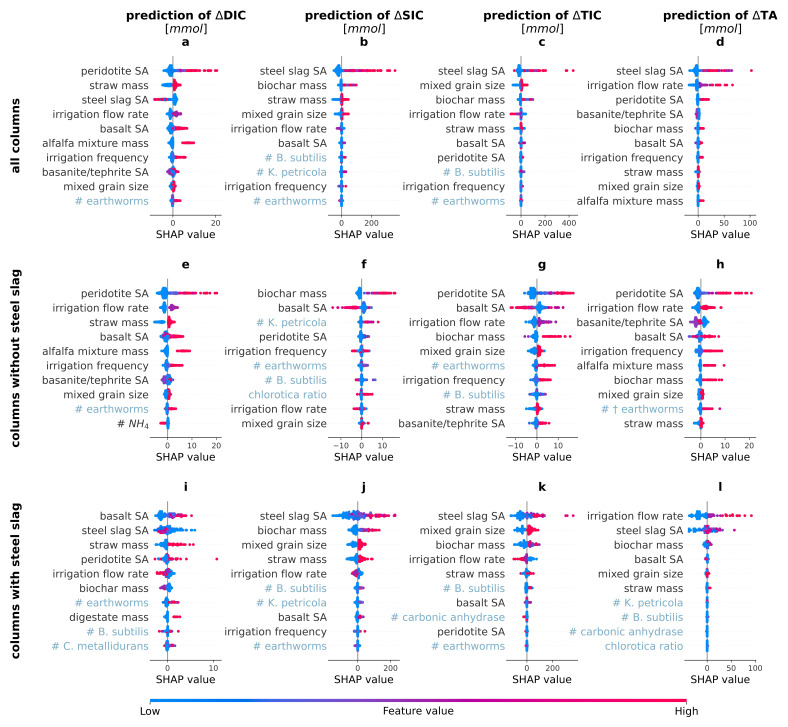
SHapley Additive eXplanation (SHAP) values of the most important drivers of the carbon dioxide removal indicators. SHAP values of the top ten most important features in the eXtreme Gradient Boosting prediction of the changes in amount of dissolved inorganic carbon (∆DIC), solid inorganic carbon (∆SIC), total inorganic carbon (∆TIC) and total alkalinity (∆TA). The plots depict a SHAP value for each prediction and show the local feature importance and the feature effect. A dot with a high SHAP value for a feature suggests a positive contribution to the prediction, whereas a negative SHAP value leads to a lower prediction. The color of a dot represents the value of the feature in that instance - red indicating relatively high, blue indicating relatively low values (For the binary features, high values = true, low values = false; for chlorotica ratio, red means 100% A. chlorotica earthworms, blue means 100% A. caliginosa earthworms). For example, a red dot with a positive SHAP value implies that a higher value of the feature elicits an increase in the target value. SA stands for surface area, # stands for added amount of biota. The features are ranked in order of descending average importance and biotic features are indicated in light blue for clarity.

**Figure 3.**
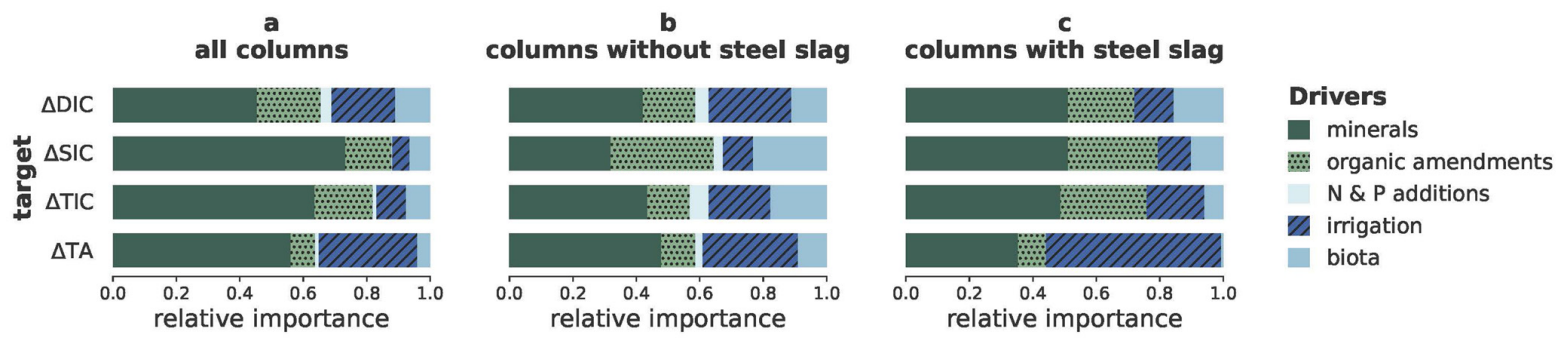
Importance of groups of features to carbon dioxide removal indicators. Relative importance of the main groups of drivers (rocks, organic amendments, irrigation, N and P additions, and biota and biotic products) to the prediction of the changes in amounts of dissolved inorganic carbon (∆DIC), solid inorganic carbon (∆SIC), total inorganic carbon (∆TIC) and total alkalinity (∆TA); Calculated using Equation 2.2.

As the model with the highest average performance across all targets ( Table 2), XGB, is a black box model, we calculated the corresponding SHAP values for the individual features ( Figure 2) and for the main groups of features ( Figure 3) to understand which of the investigated drivers contributed most to the predictions.

In agreement with our hypothesis, the SHAP values revealed that ∆SIC, ∆DIC and ∆TA were indeed driven by different features ( Figure 2, Figure 3). Disparities in the drivers of ∆TA and ∆DIC likely indicate a confounding effect of organic alkalinity production or consumption due to the added organics, steel slags or biota. Precipitation of SIC depends on several conditions (saturation, pH, availability of seed crystals ( Amann *et al.*, 2022)), and SIC can also be dissolved, leading to ∆SIC being driven by different features than ∆DIC and ∆TA. The target that is most directly related to CDR was ∆TIC, i.e. the sum of ∆DIC and ∆SIC. Its SHAP values are therefore a mix of the SHAP values of ∆DIC and ∆SIC. The next paragraphs discuss in detail the SHAP values of the dominant driver groups of the different targets.

***Rocks.*** Among all potential drivers tested in this experiment, the choice of type of rocks had the biggest impact on the predictions. For ∆DIC, adding peridotite to the rock powder mix had the largest positive effect. This is clearly shown in Figure 2-a, where the columns including peridotite (red dots of peridotite SA) all exhibited higher SHAP values than those without peridotite (blue dots), thus indicating that according to the model, adding peridotite increases ∆DIC the most. In addition to peridotite, also basalt increased ∆DIC predictions ( Figure 2-a). As the ultramafic peridotite is known to weather faster than the mafic basalt, these findings are in line with expectations ( Hartmann *et al.*, 2013).

In contrast to peridotite and basalt, adding steel slag reduced ∆DIC ( Figure 2-a). This is likely because the increased pH shifted the IC equilibrium to carbonate, which precipitated to form SIC (as is indeed seen in Figure 2-b). However, for the other CDR indicators, steel slag was identified as the most important feature, consistently increasing the predicted values ( Figure 2-b,c,d). The steel slag used had a high calcium content (Table A6 in Extended Data, XRF), a portion of which was likely present in the form of calcium hydroxide (Ca(OH) _2_). This phase dissolves quickly in water, increasing solution pH by releasing hydroxide ions. Due to the abundance of cations and the high pH, where CO _3_^2–^ is the main species of IC, the solution will be oversaturated with respect to some carbonate minerals. The precipitated CaCO _3_ can in turn serve as a seed crystal for further CaCO _3_ precipitation, thereby reducing ∆DIC and increasing ∆SIC and ∆TIC, while the formed hydroxides cause an increase in ∆TA. Interestingly, adding steel slag to the columns led to different SHAP values for some features, such as irrigation flow rate. This is visualized by the blue and red dots (indicating the absence/presence of the feature) switching sign when comparing the SHAP values of some features between the columns with and without steel slag ( Figure 2-e-,f,g versus Figure 2-i,j,k). Apparently, using steel slag introduced a suite of interactions that altered the impact of these other features, possibly due to the accompanying increase in pH.

In the absence of steel slag, columns that contained more peridotite clearly exhibited faster weathering than average, as indicated by the higher SHAP values for ∆DIC, ∆SIC, ∆TIC as well as ∆TA ( Figure 2-e,f,g,h). Columns containing more basalt also exhibited higher SHAP values for ∆DIC and ∆TA, but lower for ∆SIC. For ∆TIC, fine basalt grains (high basalt SA) resulted in lower ∆TIC and coarse grains in higher ∆TIC (medium basalt SA). Adding more basanite/tephrite to the rock mix had no clear effect on ∆SIC and a positive one on ∆TIC (Figures C3-C4 in Extended Data). Coarse grained basanite/tephrite led to a lower ∆DIC and adding basanite/tephrite had a negative effect on ∆TA, especially coarse grains. The negative SHAP values do not necessarily mean that adding the rock decreased CDR, but that it yielded smaller increases in these targets than other rocks, and its addition comes at the expense of a lower content of the other rocks.

Mixing the grain sizes increased the predictions of the CDR indicators ( Figure 2). As such, columns with mixed coarse and fine grain sizes may have had similar SA as columns composed of medium-sized grains, yet exhibited higher CDR.While finer grains typically correspond to more nucleation sites for carbonate precipitation, mixing them with coarser grains might have enhanced an efficient water flow ( Amann *et al.*, 2022).

***Irrigation.*** The irrigation flow rate and irrigation frequency were also commonly important drivers of weathering rate in the XGB models. The irrigation flow rate had opposing effects on the predicted ∆DIC and ∆TIC for the subsets of columns with and without steel slags. In columns without steel slags, higher irrigation flow rates increased predicted ∆DIC, ∆TIC and ∆TA ( Figure 2-e,g,h and Figure C6-a1,i1,m1 in Extended data). This behaviour could be expected, as a higher flow through transports more cations and bicarbonate ions into the leachates, driving the weathering reactions further from equilibrium ( Evans & Banwart, 2006; Maher, 2010). However, for ∆DIC and ∆TA, only small differences were detected between the impacts of the medium flow rate and the highest flow rate (100 and 150 ml day ^-1^; equivalent to rainfall fluxes of roughly 10000 and 15000 mm year ^-1^). Possibly, 100 ml day ^-1^ flushed sufficient dissolved base cations and bicarbonate ions from the column to maintain the solution undersaturated with respect to secondary mineral formation, with stronger dilution only marginally affecting mineral weathering. This suggests that the reactions governing ∆DIC and ∆TA are not transport-limited at flow rates of 0.25 ml day ^-1^g ^-1^ rocks (100 ml day ^-1^ per 400g rocks) or higher, and that the limit likely lies between 0.125 ml day ^-1^g ^-1^ rocks and 0.25 ml day ^-1^g ^-1^ rocks.

In the subset including only columns with steel slag, a negative response to irrigation flow rate was observed for ∆DIC, ∆SIC and ∆TIC, whereas for ∆TA, high flow rates exhibited positive SHAP values ( Figure 2-i,j,k,l and Figure C6-a2,e2,i2,m2 in Extended data). Possibly, higher flow rates transported more cations to the leachate, increasing ∆TA and lowering ∆SIC and as a result also ∆TIC. The increased pH in the leached solution due to the added steel slags could have brought the system far from equilibrium, leading to continuous precipitation of carbonates in the leachate container ( Moras *et al.*, 2022).

In addition to irrigation flow rate, also irrigation frequency was selected as an important driver, exhibiting a positive effect on the predicted ∆DIC, ∆TIC and ∆TA in columns with no steel slag. This was in line with expectations, as a higher irrigation frequency implies more continuous replacement of the saturated pore water. Higher irrigation frequencies may also have accelerated weathering by decreasing average pO _2_, possibly retarding formation of oxides that are known to decrease weathering rates by orders of magnitude ( Fuhr *et al.*, 2024; Oelkers *et al.*, 2018).

***Organic amendments and biochar.*** The organic amendments (straw, digestate and the alfalfa mixture) were added to create a livable environment for the biota. By providing the substrates used in respiration, they were expected to accelerate CDR. In agreement, adding straw to the columns increased ∆DIC, ∆SIC (when combined with steel slag), ∆TIC, and ∆TA ( Figure 2). Digestate had a positive effect on ∆DIC (Figure C2 in Extended Data), and a negative one on ∆TA (Figure C5 in Extended Data), while its other effects were negligible. The alfalfa mixture was only added to batches without steel slag and had a large positive effect on the predicted ∆DIC and ∆TA ( Figure 2-e,f,g,h) and a small negative effect on ∆SIC (Figure C3 in Extended Data), resulting in a positive effect on ∆TIC (Figure C4 in Extended Data). Its SHAP values were generally more positive than for straw, possibly because it is a better palatable organic matter source. Organic matter additions, and especially straw and the alfalfa mixture, thus seem to have increased mineral weathering, possibly due to increased CO _2_ concentrations following organic matter decomposition and increased porosity and water flow in the column.

Biochar was not added to stimulate microbial activity, but to promote weathering abiotically by adsorbing ions and increasing seed crystals due to its high IC content and pH (Table A8 in Extended Data). It indeed achieved a large positive effect for ∆SIC, ∆TIC and ∆TA ( Figure 2), although the increased SIC precipitation logically led to no net increase or even a decrease in ∆DIC (Figure C5 in Extended Data). The absence of a rise in ∆DIC suggests that the increase in ∆TA was of organic origin.

***Biota and biotic products.*** Also certain biota were identified as important drivers of the targets. The number of earthworms, for example, frequently was a positive driver. However, as a substantial part of the added earthworms died during the experiment, their effect on the targets could also be due to decomposing earthworms. To investigate this, also dead earthworms were added to certain columns. The SHAP values revealed that earthworms that were alive at the start of the experiment (# earthworms) positively impacted ∆DIC and, in absence of steel slag, also ∆SIC, ∆TIC and ∆TA, while (initially) dead earthworms (# † earthworms) positively impacted the predicted ∆DIC and ∆TA ( Figure 2).

The positive effect of (initially) live earthworms on ∆DIC and ∆TA ( Figure 2a,e,i and Figure C5 in Extended data) agrees with our expectations. Earthworms accelerate organic matter decomposition by fragmenting organic matter, consequently increasing its surface area and inoculating it with microbes in their gut ( Edwards & Arancon, 2022). Moreover, earthworms stimulate microbial activity and distribution by producing mucus and concentrating nutrients ( Amador & Görres, 2007; Brown, 1995; Frouz *et al.*, 2011; Van Groenigen *et al.*, 2019). This accelerated decomposition of organic matter and higher microbial activity leads to increased release of CO _2_ ( Ajwa & Tabatabai, 1994; Condron *et al.*, 2010), which could have enhanced mineral weathering ( Amann *et al.*, 2022) and led to an increased formation of bicarbonate in solution ( Manning *et al.*, 2024). However, as the number of (initially) dead earthworms had similar SHAP values as the number of (initally) live earthworms, the increased ∆DIC and ∆TA could also be a result of decomposing earthworm bodies, e.g., by increasing the microbial activity due to an immediate pool of available nutrients ( Kos *et al.*, 2016; Lin *et al.*, 2022; Sun & Ge, 2021). This higher microbial activity could have further increased decomposition and led to a higher CO _2_ release and consequently to ∆DIC formation through weathering ( Calogiuri *et al.*, 2025).

The positive effect of (initially) live earthworms on the predicted ∆SIC was only seen for columns in which no steel slag was present ( Figure 2f). In contrast to ∆DIC and ∆TA, the predicted ∆SIC was not substantially impacted by (initially) dead earthworms, indicating that earthworms likely actively increase ∆SIC. This could occur through physical and chemical processes happening within their bodies when earthworms feed on rock particles. First, earthworms possess calciferous glands through which they can produce calcium carbonate minerals and therefore contribute to the formation of SIC ( Briones *et al.*, 2008; Versteegh *et al.*, 2014). Second, the rock particles are ground in the earthworms’ gizzards, increasing their SA ( Suzuki *et al.*, 2003) and removing poorly weathering precipitates by abrasion. These freshly exposed mineral surfaces can then be attacked by microbes living in the earthworms’ intestines ( Carpenter *et al.*, 2007; Liu *et al.*, 2011; Zhu *et al.*, 2013) and in the surrounding environment once the particles are egested. In the case of Ca-bearing rocks, this results in an increased release of Ca ^2+^, which drives re-precipitation of ∆DIC as carbonate minerals ( Manning *et al.*, 2024; Washbourne *et al.*, 2015), thereby increasing ∆SIC. However, when steel slag was present, even in small amounts, the effect of earthworms on the predicted ∆SIC was negative, but negligible with respect to the large importance of the abiotic drivers ( Figure 2-j and Figure C6-f2 in Extended data). The positive effect of the earthworms in the absence of steelslags was maximal for ∆DIC for 8 earthworms and for ∆SIC for 4 or 8 earthworms ( Figure 2-f and Figure C6-b1,f1 in Extended data). Possibly, a higher earthworm density was detrimental for earthworm well-being and might have created conditions which led to earthworms entering a diapause state instead of feeding on the mineral particles. As ∆TIC is the sum of ∆DIC and ∆SIC, and (initially) live earthworms had a positive effect on both targets for columns with no steel slag, their presence also led to higher ∆TIC ( Figure 2-g and Figure C6-j1 in Extended data), especially for 8 earthworms.

Besides earthworms, bacteria and fungi also impacted the CDR indicators, albeit less clearly. For instance, intermediate inoculum densities of *B. subtilis* had a positive effect on ∆SIC and ∆TIC especially in columns with steel slags ( Figure 2 and Figure C6-h,i in Extended data). This is in agreement with previous studies ( Ferral-Perez *et al.*, 2020; Keren-Paz *et al.*, 2022; Kim *et al.*, 2005), that showed that *B. subtilis* can promote the formation of solid inorganic carbonates, such as calcium carbonate, through a process called microbially induced calcium carbonate precipitation during the urease-catalyzed urea metabolization. This process may increase the pH and indirectly contribute to the precipitation of CaCO _3_ ( Ferral-Perez *et al.*, 2020; Keren-Paz *et al.*, 2022; Kim *et al.*, 2005).

Other notable effects are the positive impact of *K. petricola* on ∆DIC, ∆SIC and, in absence of steel slag, ∆TIC. *K. petricola* has been demonstrated to increase olivine weathering rates by hindering the formation of iron oxides on the olivine surface ( Gerrits *et al.*, 2020). *K. petricola* forms biofilms on rock surfaces, providing a unique micro-environment that could promote the precipitation of carbonate minerals. These biofilms can trap ions and organic material, allowing carbonate nucleation to occur under specific conditions. This process could result in the deposition of carbonate minerals as part of SIC formation on or around the microbial biofilm. *A. pullulans* had a positive effect on ∆DIC, and, in columns with steel slag, on ∆SIC, while the effect on ∆SIC was a negative one on ∆SIC in absence of steel slag and no clear net effect was observed on ∆TIC and ∆TA (Figures C2-C54 and C6-c,g,k,o in Extended Data). *A. pullulans* is a versatile fungus that can produce numerous different enzymes and siderophores, which could cause the ∆DIC increase. It has the ability to biomineralize metal nanoparticles, which may be of great importance in an environment rich in metallic ions such as in an ultramafic environment, where toxic metal concentrations may accumulate in a high weathering environment ( Gostincar *et al.*, 2014; He *et al.*, 2021; Nwoko *et al.*, 2021). Since the biomineralized products are oxides rather than carbonates, the negative effect on ∆SIC was expected.

*S. variegatus* was added to test whether it could enhance weathering by releasing citric acid, which not only helps mobilize nutrients from the mineral but also creates favorable conditions for bacterial growth ( Olsson & Wallander, 1998). *C. metallidurans* was tested for its resistance to high metal concentrations and its capacity of using basalt as a source of nutrients ( Byloos *et al.*, 2017; Byloos *et al.*, 2018; Olsson-Francis *et al.*, 2010). However, in this study, the effect of *S. variegatus* and *C. metallidurans* on the predicted targets was small (Figures C2-C5 in Extended Data). Therefore, P and N were added to test for microbial nutrient limitation. However, P and urea had no significant effect on the predicted CDR indicators and NH _4_Cl even had a large negative effect on ∆DIC, ∆SIC and ∆TIC, which can be attributed to its acidic nature. Consequently, possible P and N deficits were unlikely to have limited microbial activity.

In theory, the added enzymes could play a role in EW; the overexpression of laccase has been reported to enhance quartz weathering ( Kirtzel *et al.*, 2019), hydrolysis of urea leads to CaCO _3_ precipitation ( Dilrukshi *et al.*, 2018; Krajewska, 2018), and carbonic anhydrase catalyzes the conversion of CO _2_ and H _2_O to bicarbonates and protons (or reverse), therefore potentially accelerating the supply of protons to weather the minerals ( Peplow, 2024; Watson *et al.*, 2016). In contrast, in this experiment, addition of these three enzymes had negligible, and even negative effects on the predicted CDR indicators.

### Limitations

This study also faced limitations, notably the imperfect representations of CDR indicators used, which may have been affected by the open system and unmeasured particulate carbon accumulation on funnels and tubes. In addition, ∆TA, ∆DIC and ∆TIC were calculated using the irrigated volume and not the volume remaining in the leachate containers, as these volumes did not agree. We assumed all irrigation water passed through the columns and therefore that the volume lost contained the same concentrations. The reader should also bear in mind that the identified drivers that dominate weathering in this experiment may differ from those dominating CDR in the long term. Moreover, this experiment was conducted in the absence of soil to test optimal conditions in reactor settings, which may also have affected the relative importance of the drivers compared to field studies in soils.

Additionally, while the ML and xAI methodology provided valuable insights, they are correlation-based rather than causation-based, which should be considered when interpreting these findings. Last, the nested CV approach provides a robust estimate of model generalization performance and is appropriate for our objective, i.e. uncovering insights from driver importance. However, future applications aimed at model deployment should include evaluation on a separate hold-out test set reserved prior to model development.

Future work should focus on refining these experimental setups and exploring more direct measurements of CDR to quantify the potential of enhanced weathering as a viable CDR technology.

## Conclusion

This study presents an extensive enhanced weathering experiment designed to explore how various abiotic and biotic factors influence CDR. ML allowed prediction of key weathering indicators (∆ *DIC*, ∆ *SIC*, ∆ *TIC* and ∆ *TA*) in a highly uneven dataset. The integration of SHAP value analysis revealed that the four weathering indicators are driven by different features. Nonetheless, the rock-related features consistently dominated all predicted targets. In particular, the addition of steel slags, and to a lesser degree peridotite and basalt, significantly increased weathering rates. Moreover, steel slag introduced complex interactions that led to varying effects on carbon sequestration. This interaction was especially clear in the impact of the irrigation regime. While higher water throughput rates and more frequent irrigation generally increased predicted weathering indicators, higher throughput rates led to lower ∆ *DIC*, ∆ *SIC*, ∆ *TIC* for columns with steel slag. Besides the choice of rocks and irrigation, also biochar and organic amendments, in particular straw and alfalfa-dominated mixtures, positively impacted CDR. Last, earthworms, bacteria and fungi had an impact on the weathering rate, often being one of the top ten drivers of the weathering indicators. However, the effects of the microbes varied depending on the predicted target and on the presence of steel slag. In conclusion, this research underscores the complexity of enhanced weathering processes, offers important insights for future efforts in utilizing enhanced weathering as a carbon removal strategy and demonstrates the potential of ML and xAI in uncovering key drivers of complex biochemical systems.

## Ethics and consent

 Ethical approval and consent were not required.

## Data Availability

The column experiment data are produced as part of the Bio-Accelerated Mineral weathering (BAM) project. No data will be released during the project (even upon request) to enable relevant publications or patent filings. In accordance with the BAM project’s Data Management Plan, all data will be made openly accessible latest three years after the project’s conclusion (2028). At that time, the underlying data repository will be specified on Zenodo: Extended data for ‘Machine learning-based identification of key biotic and abiotic drivers of mineral weathering rate in a complex enhanced weathering experiment’, https://www.doi.org/10.5281/zenodo.16728794 ( Janssens *et al.*, 2025). Zenodo: Extended data for ‘Machine learning-based identification of key biotic and abiotic drivers of mineral weathering rate in a complex enhanced weathering experiment’, https://doi.org/10.5281/zenodo.16728794 ( Janssens *et al.*, 2025). The project contains the following extended data: Extended info.pdf ( information about the used materials (A), about the data processing and additional noise analysis (B), and about the machine learning analysis and extended Shapley Additive exPlanation figures (C)). Data are available under the terms of the Creative Commons Zero "No rights reserved" data waiver (CC0 1.0 Public domain dedication)( http://creativecommons.org/publicdomain/zero/1.0/).

## Software availability

Analysis code available from Github: https://www.github.com/STAN-UAntwerp/h2020_bam

Archived software available from: https://doi.org/10.5281/zenodo.14528497 ( Janssens *et al.*, 2024)

Software is available under the MIT License
