# Supplementary material for: Machine learning-based identification of key biotic and abiotic drivers of mineral weathering rate in a complex enhanced weathering experiment
Source: Open Res Eur. 2025 Sep 11;5:71. Originally published 2025 Mar 18. [Version 3] doi: 10.12688/openreseurope.19252.3 (PMC12338168; doi:10.12688/openreseurope.19252.3)
Supplement: Peer review history

# Reviewer report: Reviewer response for version 3

**DOI:** 10.21956/openreseurope.22857.r59934

**Published:** 2025-09-23

**Authors:** Xiying Sun

**Affiliations:** 1 Texas A&M University, Texas, USA

I would like to thank the authors for their thorough revisions. They have successfully addressed all of the concerns I raised in my previous review. The manuscript is now a much stronger and more reproducible piece of work. I am pleased to recommend this article for full approval and have no further comments.

Is the study design appropriate and does the work have academic merit?

Yes

Is the work clearly and accurately presented and does it cite the current literature?

Yes

If applicable, is the statistical analysis and its interpretation appropriate?

Yes

Are all the source data underlying the results available to ensure full reproducibility?

No

Are the conclusions drawn adequately supported by the results?

Partly

Are sufficient details of methods and analysis provided to allow replication by others?

No

Reviewer Expertise:

NA

I confirm that I have read this submission and believe that I have an appropriate level of expertise to confirm that it is of an acceptable scientific standard.

# Reviewer report: Reviewer response for version 2

**DOI:** 10.21956/openreseurope.22471.r56415

**Published:** 2025-07-26

**Authors:** David Manning

**Affiliations:** 1 Newcastle University, Newcastle upon Tyne, UK

Thank you for addressing my suggested changes so carefully.  The Supplementary Information is really informative and would allow other scientists to use this study in a substantial and serious way.

 I’d like it if you could rethink some text in the penultimate paragraph of the Introduction: ‘implying that the same drivers would induce similar changes in both, albeit with opposite signs.’  This is not necessarily so. If calcite (SIC) as the solution are in equilibrium, the concentration of DIC should be constant and will only decrease once calcite has dissolved completely.  Precipitation of more calcite will maintain constant [DIC].

 Relating to this, in the results section ‘Organic Amendments and Biochar’, the statement ‘The absence of a rise in ∆DIC suggests that the increase in TA was of organic origin’ may be wrongly interpreted, as it could instead reflect calcite saturation.  Geochemical modelling of the solution composition would tell you this. Can you rule out calcite saturation?  If so, please say so.

Is the study design appropriate and does the work have academic merit?

Yes

Is the work clearly and accurately presented and does it cite the current literature?

Yes

If applicable, is the statistical analysis and its interpretation appropriate?

Partly

Are all the source data underlying the results available to ensure full reproducibility?

No

Are the conclusions drawn adequately supported by the results?

Partly

Are sufficient details of methods and analysis provided to allow replication by others?

No

Reviewer Expertise:

Soil science, mineralogy, petrology, biochar

I confirm that I have read this submission and believe that I have an appropriate level of expertise to confirm that it is of an acceptable scientific standard.

# Response

**Published:** 2025-08-02

**Authors:** Iris Janssens

**Affiliations:** Computer science, Universiteit Antwerpen Faculteit Wetenschappen, Antwerp, Flanders, Belgium

Thank you for your detailed feedback, which has been invaluable in enhancing the quality of our manuscript.

- We agree with the referee: if a solution is at equilibrium, adding IC to it would increase SIC with constant DIC. However, we had in mind driver-induced changes relative to the controls. Take the example of using steel slag as mineral substrate instead of basalt. Its weathering increases pH so strongly that all formed IC (released from added substrates or by heterotrophic respiration in the columns) is immediately precipitated as calcite. In this case, as the referee suggests, inside the pot DIC remains constant (in fact zero), while SIC increases strongly. However, compared to the starting concentrations and to the pots with basalt, DIC declines and SIC increases. Another example: adding a chelate would mask part of the DIC, effectively increasing the equilibrium constant at which calcite precipitates, and thereby increasing DIC and decreasing SIC precipitation. To avoid similar criticisms by the readers, we rephrased the sentence as: “ *Moreover, in a purely abiotic system, DIC and SIC are in equilibrium (yet controlled by kinetic rate constants of carbonate precipitation and dissolution), implying that a driver that accelerates or decelerates SIC formation would induce opposite changes in DIC and SIC, relative to comparable columns without the driver.*”
- Indeed, the absence of a rise in ∆DIC may come from calcite precipitation, but this could not explain why ∆TA increased simultaneously, because calcite precipitation is an alkalinity sink. So if calcite saturation were at play, both ∆TA and ∆DIC should decline with increasing ∆SIC, which we did not observe. The three figures below show for three different ∆DIC ranges the corresponding ∆SIC and ∆TA values, revealing no correlations and suggesting that TA can change substantially also in absence of large values of ∆SIC or ∆DIC. A manuscript dedicated to the importance of the organic fraction of TA using a subset of this dataset and showing clear evidence for a major contribution of organic alkalinity has been submitted for publication elsewhere. Unfortunately, that manuscript is still under review so we cannot give a definite citation for it, but together with the absence of correlation with ∆SIC, it does rule out that carbonate saturation is causing these observations. 

​​​​​​​Fig- https://openreseurope-files.f1000.com/linked/258401.Fig_2_19252.png

# Reviewer report: Reviewer response for version 2

**DOI:** 10.21956/openreseurope.22471.r56413

**Published:** 2025-07-15

**Authors:** Xiying Sun

**Affiliations:** 1 Texas A&M University, Texas, USA

The authors have done an excellent job addressing most of my previous concerns. The added details on the input materials and the new machine learning results in the Extended Data have substantially strengthened the manuscript.

 The paper is very close to being ready for full approval. However, I recommend a few final revisions to ensure the analysis is fully transparent and reproducible.

 1. SHAP interaction plots: To directly visualize the conditional effects that are central to the paper's conclusions, I still strongly recommend including SHAP interaction plots. As the authors are already using the shap library in Python, this can be implemented with the standard shap.dependence_plot() function and should not require significant additional work.

 2. Justification for data omission: My previous review raised a major point about the potential bias from omitting data from rounds 1-3 for some targets. This remains unaddressed. A brief discussion of this choice and its potential limitations should be added to ensure the methodology is fully transparent.

 3. Correction of potential error in Extended Data: The newly added "Extended Data" file is very helpful. However, potential error is in Table A6, where the error reported for SO3 in basalt is ±1.00% on a value of 0.01%, which is scientifically implausible. 

 For these reasons, I recommend maintaining the status of Approved with reservations. I am confident that if the authors address these points, I will be able to recommend the article for full approval.

Is the study design appropriate and does the work have academic merit?

Yes

Is the work clearly and accurately presented and does it cite the current literature?

Yes

If applicable, is the statistical analysis and its interpretation appropriate?

Yes

Are all the source data underlying the results available to ensure full reproducibility?

No

Are the conclusions drawn adequately supported by the results?

Partly

Are sufficient details of methods and analysis provided to allow replication by others?

No

Reviewer Expertise:

NA

I confirm that I have read this submission and believe that I have an appropriate level of expertise to confirm that it is of an acceptable scientific standard, however I have significant reservations, as outlined above.

# Response

**Published:** 2025-08-02

**Authors:** Iris Janssens

**Affiliations:** Computer science, Universiteit Antwerpen Faculteit Wetenschappen, Antwerp, Flanders, Belgium

We sincerely thank you for the in depth review that helped us strengthen our manuscript.

1. We have now added several SHAP interaction plots to the Extended data (figure C6) and added references to these plots to the discussion. This indeed added clarity to some of the discussion points.
2. Sorry for this, we misunderstood your previous question and provided a response explaining why it was OK to omit them instead. The justification for omitting these three rounds was given in the second paragraph of the section on data pre-processing: “In rounds 1 to 3, additional samples were taken from the leachate after three weeks, which affected the measurements after eight weeks. Therefore, data of rounds 1 to 3 were omitted for the prediction of ΔTA, ΔDIC and ΔTIC.” However, we now see that the justification for the omission and its potential introduction of bias in the dataset indeed needed strengthening. We therefore elaborated the sentence as follows: "In rounds 1 to 3, additional samples for chemical analyses were taken from the leachate after three weeks, which affected the measurements after eight weeks. Therefore, data of rounds 1 to 3 were omitted for the prediction of ΔTA, ΔDIC and ΔTIC. Because the leachate sampling happened in the leachate container, it did not impact the column with solid matter above, whose data were still used. As we realized early in the project that the leachate data from the first three rounds could not be used in this analysis, many input combinations covered in these first three rounds were repeated in later rounds. As a result, this information was not lost for the ∆DIC, ∆TA and ∆TIC models. While the ∆SIC data contained more replicates, these were properly dealt with in the cross validation (CV) approach (see next section, 'Predictive models') to avoid bias in our analyses. Therefore, we believe that excluding the first three rounds did not introduce biases in this study. "
3. Thank you for spotting this. This was indeed wrong, and should be 0.01%. We have changed this in the Extended Data.

# Reviewer report: Reviewer response for version 1

**DOI:** 10.21956/openreseurope.20839.r53984

**Published:** 2025-06-14

**Authors:** Nate Looker

**Affiliations:** 1 Stanford University, Stanford, USA

This manuscript presents new analyses of a high-dimensional enhanced mineral weathering experiment, a portion of which was previously analyzed in Calogiuri et al. (2025). The complex study design allows exploration of the response of weathering rates (inferred using DIC, SIC, and TA) to 29 independent variables. The inclusion of several biological variables is an important distinction from previous lab-based enhanced weathering studies, as soil-dwelling organisms and biologically derived compounds (e.g., enzymes and organic acids) may play underappreciated roles in determining in-field weathering rates.

 Overall, the study is well designed and executed. My primary recommendation is that the authors further leverage the design and statistical analyses to deliver more interpretable insights. For example, what design-based comparisons can be made, if any? I understand that the design was not full-factorial (i.e., treatments represent a subset of all possible combinations of factor levels), but of the treatments that were explored, are there subsets that can be analyzed as varying in only one variable (e.g., mineral grain size, presence/absence of a particular additive)? Additionally, I was left wondering what the three sets of models can tell us about interactions (e.g., effect of earthworm biomass as mediated by mineral feedstock type) and the functional form (or at least direction) of effects. The Lasso regression models did not perform as well as the more sophisticated ML models, but did Lasso-estimated coefficients agree with the direction of effects inferred with ML? Partial dependence plots (as the authors previously presented in Calogiuri et al. 2025) or bivariate SHAP plots could be effective ways to explore these questions. I also encourage the authors to consider including a conceptual model to synthesize their hypotheses and/or results (e.g., expanding the earthworm-specific model in Calogiuri et al. 2025 to include the other variables analyzed here).

 Specific comments:

 Portions of the introduction (starting with the fifth paragraph on page 5) present study-specific details that would fit better in the methods section. Consider instead using the introduction to review the state of the science of interactive/nonlinear controls on enhanced weathering (i.e., the motivation for a complex study like this).

 Hypotheses (page 6): different features were hypothesized to drive change in each of the weathering indicators—can you get more specific about which features you expected to drive each indicator?

 How were treatments allocated temporally (across rounds)?

 Was autoclaving one of the 29 treatments (page 7)?

 How long and at what temperature were samples combusted for LOI? How was removal of organic C verified?

 The text states that leachate concentrations were calculated using irrigated volume (which to me means the volume of water applied), rather than the leachate volume: is this correct? Does this differ from the approach the authors used previously (Calogiuri et al. 2025)?

 What processes gave rise to missing data? If some analytes were “missing-not-at-random” (e.g., related to treatment outcomes), omitting columns with missing data could introduce bias.

 How sensitive are the effects of the enzymes studied here to pH?

Is the study design appropriate and does the work have academic merit?

Yes

Is the work clearly and accurately presented and does it cite the current literature?

Yes

If applicable, is the statistical analysis and its interpretation appropriate?

Partly

Are all the source data underlying the results available to ensure full reproducibility?

No

Are the conclusions drawn adequately supported by the results?

Yes

Are sufficient details of methods and analysis provided to allow replication by others?

Yes

Reviewer Expertise:

Soil carbon cycling, pedology, forest soils, biogeochemistry

I confirm that I have read this submission and believe that I have an appropriate level of expertise to confirm that it is of an acceptable scientific standard, however I have significant reservations, as outlined above.

# Response

**Published:** 2025-06-23

**Authors:** Iris Janssens

**Affiliations:** Computer science, Universiteit Antwerpen Faculteit Wetenschappen, Antwerp, Flanders, Belgium

We thank the referee for the insightful suggestions, which helped to improve the quality of the manuscript.

- As the number of possible combinations of treatments was extremely large (see all features and used amounts in Table 1), it was impossible to test them all in a full factorial design. The experimental design was therefore set up with the machine learning approach in mind. As a result, for many combinations the number of similar combinations with only one different variable is limited. For simple combinations (for example, containing no biotic inputs, but instead only one type of mineral (with one grain size), organic matter and biochar), such subsets can be extracted. Other studies have been -or are being- conducted using subsets of the data, for example Calogiuri et al. (2025), which indeed provide more specific results. However, the objective of this study is to test whether machine learning can be leveraged to maximally extract information from the entire dataset. This way, potential interactions between treatments are not excluded.  
- We agree that more results on the interaction effects and functional forms would be interesting.  
  - The direction and size of the effects is given by the SHAP plots (Figure 3). If a red dot (= adding (more of) the input) has a positive SHAP value, it means that the target is positively impacted by adding the input; if it has a negative SHAP value, adding the input decreases the impact.  
  - The models don’t give functional forms, except from Lasso, which only performed reasonably well for ∆DIC. Its coefficients are given in the Extended data (section C2). Comparing these coefficients with the SHAP plots of the XGB model (Figures 2, C2 in Extended data), we see that the most important features generally agree (e.g. dunite has the largest positive effect in both models), with slight deviations in the rankings (e.g. alfalfa mixture mass has a large positive effect in both models, but is ranked second by Lasso and sixth by SHAP). The latter can be explained by a difference in the meaning of the rankings: the rankings in the SHAP plots do not only include the size of the importance, but also the abundance of this value in the data set. However, there also are some differences. For example, urea is the sixth most important feature in the lasso model, while it has no impact in the XGB model.  
  - Other kinds of plots, such as partial dependence plots and SHAP dependence plots, can indeed indicate interactions between two features. We have studied these SHAP dependence plots but only found clear interactions with steel slag surface area. However, as we had already calculated and plotted the SHAP values separately for the subsets with and without steel slag, these dependence plots did not give additional information. Other interactions, though possibly present, were unclear in the dependence plots. 
- We do agree with the referee that a conceptual model would be beneficial. However, due to the absence of direct CDR data, which is now circumvented by looking at four CDR indicators, and the fact that the importances of the 30 investigated drivers differ across targets and in some cases depend on interactions with other drivers, it is at this point extremely difficult to extract a full conceptual model that describes the whole system. However, other studies focus on subsets of the data to develop conceptual models about specific aspects. One of this studie is Calogiuri et al., 2025, but more studies are still in progress. 

Specific comments:

- Thank you for suggesting this. We revised the first sentences of the fifth paragraph to make it better suited for an Introduction section, but did not move the paragraph because it introduces the rationale for selecting the chosen variables. Moreover, we did elaborate the third and seventh paragraphs, citing new papers on the interactions between minerals and biota that introduce non-linearities in the weathering rates. 
- This is an interesting suggestion, but after internal discussion we opted not to adopt it. Our main reasons are: 1. The requested indicator-specific hypotheses are the subject of targeted studies in preparation and 2. This manuscript focuses on the leverage potential of machine learning to identify key drivers in the presence of a multitude of possibly interacting features. By shifting towards indicator-specific mechanisms, we fear that the focus on machine learning leverage would be lost. We hope the referee will understand. 
- Treatments were predominantly chosen by the researchers in the project, either randomly or based on hypotheses and interests which changed through time based on intermediate results. Some subsets were also computationally determined, amongst others randomly through Latin hypercube sampling, to fill the input space as much as possible, or through optimal experimental design. This was used in later rounds and is the subject of a separate manuscript in preparation. Last, previously failed experiments were often repeated in a later round. Thus, determining the treatments was done through a mix of different methods, which could change over time. However, as the possible ‘ingredients’ were determined at the start of the project (with the exception of initially dead earthworms), all rounds had comparable input combinations. 
- Yes, the number of treatments was determined as the number of features in the models, and whether autoclaving was done or not was given by the feature ‘sterilized’ (see Tab. 1). However, your question made us realize that the number of treatments mentioned (29) did not agree with the number of features (30; Tab. 1), so we changed this to 30 in the text. 
- Samples were combusted for three hours at 550°C. This protocol is standard and was therefore not verified by alternative methods. We added this information to the Methods section. 
- Indeed, your interpretation is correct. We observed that the leachate volume did not consistently agree with the irrigated volume, which could have been due to different evaporation from containers across placements in the climate chamber, leakage after passing through the column, and small deviations of the irrigation system. However, most likely, most of these discrepancies originated after passing through the column, which would indicate that the irrigation volume is a more accurate quantity of the volume that passed through the system. Moreover, this also meant that using the irrigated volume to calculate the amounts of DIC [mmol] and TA [mmol] from their measured concentrations would give a more accurate estimation than using the leachate volume. This was added to the Limitations subsection of the manuscript. 
- Missing data could have different reasons. Sometimes errors in measurements of SIC, DIC and TA lead to the experiments being discarded for the corresponding target. Sometimes the experiment failed for some columns due to the irrigation system malfunctioning, a leak in the system, flooding of columns and human errors in the set up and during the experiment. The flooding of columns was investigated, as it could be related to the inputs, but no pattern was found. Moreover, as the experiment was conducted in different rounds, many of the failed experiments were successfully repeated in a later round. We are therefore confident that our dataset did not show a bias due to ‘not random missing data’. We have now clarified in the manuscript that the missing target data was due to measurement errors.
- The following figure plots the SHAP values of the concentrations of the three enzymes as a function of the pH of the leachate. Generally, the SHAP values are or are close to zero, and no pH effect is observed.

Figures- https://openreseurope-files.f1000.com/linked/258400.Fig_19252.png

# Reviewer report: Reviewer response for version 1

**DOI:** 10.21956/openreseurope.20839.r53989

**Published:** 2025-05-27

**Authors:** Xiying Sun

**Affiliations:** 1 Texas A&M University, Texas, USA

This study represents an ambitious and carefully designed contribution to understanding the complex drivers of enhanced weathering. The combination of a multifactorial experimental setup with machine learning provides a promising approach to disentangle the roles of biotic and abiotic variables in carbon transformation processes. The methodology is well-matched to the complexity of the system, and the findings are likely to be of broad interest within the fields of biogeochemistry and carbon dioxide removal. However, there are some important areas where clarification and additional detail would strengthen the manuscript.

 Major points requiring attention include:

1. The term “basalt (basanite), lava (basanite)” is unclear since basalt is a type of lava (solidified), are they from different sources? Do they have different textural properties despite both being basanite (e.g., one is more vesicular, hence broadly termed “lava”, while the other is a more standard “basalt”)? I recommend the authors improve the clarification and provide detailed chemical characterization.
2. The feature “# earthworms” reflects the number of initially live earthworms (Table 1). However, the manuscript acknowledges that “a substantial part of the added earthworms died during the experiment”. As a result, the SHAP importance for this feature in Figure 2 and discussed in the “Biota and biotic products” subsection likely captures both the effects of live worm activity (e.g., mineral grinding, CaCO3 production) and post-mortem decomposition (e.g., CO2 and nutrient release). Attributing the entire SHAP signal to active biological processes may therefore be misleading. I recommend clarifying this point and reconsidering the interpretation of this feature’s contribution to carbon cycling outcomes.
3. The manuscript explains the reason for omitting rounds 1-3 from the ∆TA, ∆DIC, and ∆TIC targets due to intermediate sampling affecting 8-week measurements. However, it does not explicitly address the potential biases this exclusion may introduce. Since rounds 1-3 account for a substantial portion of the experimental timeline and may contain distinct early-phase weathering dynamics or unique input combinations, their omission could limit the model’s ability to capture the full variability of system behavior. This could affect both model generalizability and the interpretation of driver importance for key CDR indicators. I recommend the authors explicitly discuss these possible limitations and qualify any conclusions drawn for these targets accordingly. Also, why were these rounds not omitted for ∆SIC? If leachate sampling affected the system, it could potentially influence SIC precipitation/dissolution as well by altering solution chemistry.
4. While Extended Info B (Table B1) helpfully lists the names of tuned hyperparameters for RF and XGBoost, the manuscript would benefit from including the full value grids used during tuning and a summary of the selected optimal values for each outer fold. Additionally, adding predicted versus actual scatter plots for the best-performing models, particularly XGBoost, would provide valuable visual insight into model fit and error structure. Including these elements, even in the supplementary materials, would enhance reproducibility of the machine learning workflow.

Some minor points for consideration:

1. The manuscript’s current use of SHAP value comparisons across subsets (e.g., with vs. without steel slag) offers useful insights into potential feature interactions. The paper would benefit further from incorporating SHAP interaction plots (plotting a feature's SHAP value against its value, colored by an interacting feature), which directly visualize how the effect of one feature on model predictions varies depending on the value of another. This would strengthen the interpretation of conditional effects already discussed in the text, such as the differing impacts of irrigation flow rate or biotic inputs depending on the presence of steel slag or organic amendments. Including a few targeted SHAP interaction plots in the supplementary materials could enhance the clarity and depth of the paper’s explainable AI analysis.
2. The “mixed grain size” feature is marked as important, but its definition and distinction from the detailed surface area (SA) features are unclear. Since SA already reflects grain size composition, it is not evident what unique information this feature adds. To clarify its role, the manuscript would benefit from addressing: (1) Does “mixed grain size” refer to mixing within a single mineral or across different minerals? (2) What does it capture that SA features do not-such as effects on column packing or flow? Clarifying these points would help ensure the SHAP importance of this feature is not misinterpreted or confounded with other inputs.
3. Extended Info B includes SHAP plots for XGBoost across most targets and for RF only in the case of ∆TA. Since RF performed comparably to XGBoost for several outcomes, the manuscript would benefit from a brief comparison of SHAP feature importance rankings between the two models for another key target such as ∆TIC. Including this in the supplementary material could strengthen confidence in the robustness of the identified drivers.
4. A small typo in “the concentrations of and TA” in the Method section.
5. The caption for Figure 1 explicitly states it includes histograms for “dissolved organic carbon (DOC)”. However, DOC is not displayed in any of the Figure 1 panels. Also, the “five targets” should be four accordingly.
6. While the study offers a valuable snapshot of early-stage mineral weathering dynamics, the temporal scope constrains conclusions about the durability of carbon sequestration and the evolving role of key drivers over time. A discussion of these limitations, particularly regarding the permanence of ∆TIC and the potential shifts in driver importance beyond the experimental window, would help appropriately qualify the interpretation of “CDR potential” and provide clearer context for future studies aiming to translate lab-scale findings to field-scale applications. For discussion on the long-term sequestration potential of DIC and its fate in natural systems, please refer to Beerling et al. (2020)
7. The use of nested cross-validation in this study is appropriate and provides a robust estimate of model generalization performance. However, if the authors intend to deploy or highlight a final, single predictive model beyond the evaluation framework, it would be ideal to validate that model on a separate hold-out test set that was set aside prior to any model tuning or training. While not essential for the study’s current focus on driver importance and methodological insights, clarifying this point or outlining such a step for future applications would strengthen the reproducibility and potential downstream utility of the modeling approach.
8. “Biochar was not amended to stimulate microbial activity, but to promote weathering abiotically by adsorbing ions and increasing seed crystals due to its high IC content and acidification of the system.” Biochar is generally alkaline and tends to increase soil pH (Rashid et al., 2020), so the observed acidification in the presence of biochar should be explicitly explained.

References

 Beerling, D. J., Kantzas, E. P., Lomas, M. R., Wade, P., Eufrasio, R. M., Renforth, P., et al. (2020). Potential for large-scale CO2 removal via enhanced rock weathering with croplands. *Nature*, *583*(7815), 242–248. https://doi.org/10.1038/s41586-020-2448-9

 Rashid, M., Hussain, Q., Khan, K. S., Al-Wabel, M. I., Afeng, Z., Akmal, M., et al. (2020). Prospects of Biochar in Alkaline Soils to Mitigate Climate Change. In S. Fahad, M. Hasanuzzaman, M. Alam, H. Ullah, M. Saeed, I. Ali Khan, & M. Adnan (Eds.), *Environment, Climate, Plant and Vegetation Growth* (pp. 133–149). Cham: Springer International Publishing. https://doi.org/10.1007/978-3-030-49732-3_7

Is the study design appropriate and does the work have academic merit?

Yes

Is the work clearly and accurately presented and does it cite the current literature?

Yes

If applicable, is the statistical analysis and its interpretation appropriate?

Yes

Are all the source data underlying the results available to ensure full reproducibility?

No

Are the conclusions drawn adequately supported by the results?

Partly

Are sufficient details of methods and analysis provided to allow replication by others?

No

Reviewer Expertise:

Oceanography, Machine Learning, Carbon Cycle, Silicate Weathering

I confirm that I have read this submission and believe that I have an appropriate level of expertise to confirm that it is of an acceptable scientific standard, however I have significant reservations, as outlined above.

## References

[1] : Potential for large-scale CO2 removal via enhanced rock weathering with croplands. *Nature* .2020;583(7815) : 10.1038/s41586-020-2448-9 242-248 10.1038/s41586-020-2448-9 32641817

[2] : Prospects of Biochar in Alkaline Soils to Mitigate Climate Change.2020; 10.1007/978-3-030-49732-3_7 133-149 10.1007/978-3-030-49732-3_7

# Response

**Published:** 2025-06-23

**Authors:** Iris Janssens

**Affiliations:** Computer science, Universiteit Antwerpen Faculteit Wetenschappen, Antwerp, Flanders, Belgium

Thank you for the thorough evaluation and the constructive suggestions that helped us improve the manuscript. Major points:

1. The rocks were indeed not properly described. We have renamed lava to basanite/tephrite (and dunite to peridotite) and added a clearer explanation to the methods. Moreover, we added a new section (A) to the Extended Data, containing amongst others a TAS diagram and XRF and XRD results for the rocks (section A.2).  
2. We indeed realized that earthworms died during the experiment. To distinguish the impacts of living versus decaying earthworms, we also conducted experiments where only dead earthworms were added. Then, we always compared the SHAP values of the initially live earthworms with the initially dead earthworms and interpreted the results accordingly. This is discussed in the 'Biota and biotic products' subsection.
3. To answer you last question; the data of the first three rounds could be used for the ∆SIC models, because the leachate sampling happened in the leachate container, which did not impact the column with solid matter above. To adress your first point; as we realized early in the project that the first three rounds would not be used in this analysis, many of the input combinations covered in these three rounds were repeated in later rounds. As a result, this information was not lost for the ∆DIC, ∆TA and ∆TIC models, while the ∆SIC data contained more replicates, which were properly dealt with in the train test splits. Based on this, and the fact that the amount of data after omitting the first three rounds remains large, we believe that this exclusion did not introduce biases in the data sets.  
4. We agree that this enhances the reproducibility. I have expanded the hyperparameter table to include the selected values in each fold of each of the models (Table C1); and added the scatter plots to the Extended data (Fig C1). 

Minor points:

We agree that in many cases such interaction plots are very informative. However, we looked at these plots, and besides the interaction with steel slag, no clear interactions were found. Since these interactions with steel slag were already clear in the summary plots, we opted not to provide redundant display items.  
(1) The mixed grain size refers to both. Whether a coarse mineral A was mixed with a fine mineral B, or a fine mineral A with a coarse mineral B, is reflected in the respective total SA of minerals A and B; therefore this information was captured by the SA. However, information on mixing or no mixing needed to be provided because: (2) Whether the grain sizes were mixed or not provides additional information to the SA, both within the same rock type and across mixed rock types. For example, mixing minerals with a high and a low SA can yield a similar SA as minerals with a medium SA, but combining large and small grains can have a big impact on the water flow (Amann et al., 2022).  
  We have clarified this in the revised manuscript in the 'Rocks' subsection of the 'Results and discussion' section. 
We agree that comparing the SHAP values of RF with those of XGB strengthens the robustness and have added these plots for all targets to the Extended Data (Figs. C6-C9). The SHAP values of both models generally agree: the direction of the effects consistently agree, though there are small changes in sizes of impacts and as a result in the rankings. 
Thank you for spotting this. This is now corrected. 
This was indeed a wrong version of the caption and is corrected now.  
We agree with the referee that our results are only valid for the first 8 weeks of weathering and should not be extrapolated in time nor to field applications. We added the following text to the Limitations subsection: “The reader should also bear in mind that the identified drivers that dominate weathering in this experiment may differ from those dominating CDR in the long term. Moreover, this experiment was conducted in the absence of soil to test optimal conditions in reactor settings, which may also have affected the relative importance of the drivers compared to field studies in soils.
Thank you for this suggestion. We agree that clarifying this can be useful and we have added the following sentences to the Limitations subsection: “The nested CV approach provides a robust estimate of model generalization performance and is appropriate for our objective, i.e. uncovering insights from driver importance. However, future applications aimed at model deployment should include evaluation on a separate hold-out test set reserved prior to model development.” 
Thank you for spotting this. This was indeed a mistake in the text. The pH, measured in CaCl2 was 8.6 (this was added in a new table A8 in the extended data). The text is now corrected to: “Biochar was not added to stimulate microbial activity, but to promote weathering abiotically by adsorbing ions and increasing seed crystals, due to its high IC content and pH  (see Table A8 in Extended data).”   

# Reviewer report: Reviewer response for version 1

**DOI:** 10.21956/openreseurope.20839.r53014

**Published:** 2025-05-09

**Authors:** David Manning

**Affiliations:** 1 Newcastle University, Newcastle upon Tyne, UK

This manuscript reports a really nice piece of work that addresses a fundamental problem associated with experiments that investigate enhanced rock weathering – how do you work with so many variables.  I am not an expert in AI, but I can see the value of the approach taken here, and I find it exciting.  I am confident that once the work is indexed, it will be widely regarded as a pioneering paper that will be widely cited, the approach being emulated and developed by many other researchers.

 I always look at the supplementary material, Extended Data in this case, to see if there is enough information to allow others to repeat the experiments. In this case insufficient information is made available either in the Extended Data or in the paper that describes the experimental work, Calogiuri et al 2023.  The following information must be provided, and its disclosure will have no effect on the project’s protection of intellectual property:

1. The rocks used MUST be properly described in the Methods section.  A basanite is as different from a basalt as a lion is from a tiger.  You must use the International Union of Geological Sciences’ classification of volcanic rocks, published by Le Bas et al many years ago: Refer:1. Here you will see that a basanite has a different chemical composition to a basalt. At the very least, the chemical analysis of the rocks and slag used should be provided in the Extended Data in the conventional way (as oxides with the Loss on Ignition and the Sum), together with the location that these materials came from (or the name of the company that supplied the slag).  In each case, a reference should be cited to geological literature that describes the rocks in more detail – there will be plenty to choose from. Similarly, ‘dunite’ has a precise definition (>90% olivine), and the source should be stated again with a reference to its description.  You need to take similar care to that taken when describing the earthworms or the microbes.
2. Clearly explain what is the difference between the basanite that is a ‘basalt’ and the basanite that is a ‘lava’.  What is written in the Methods section (page 7, top left) is meaningless as it stands, and will only confuse a reader who only knows a little petrology.
3. Similarly, the biochar needs to be described in more detail, as different biochar feedstocks (e.g. wheat straw, softwood etc) lead to very different properties.  Please state what the feedstock is, and provide a reference that gives the basic properties of biochar prepared from that feedstock.
4. And in Table 1, the type of straw needs to be stated.
5. The sand: again, give a full description of the sand, in terms of its chemical and mineralogical composition and its source. Sand is a really variable material. It often contains calcite and aragonite as shells, and it often contains feldspars, rock fragments and other confounding components.
6. The water used needs to be clearly described. I don’t think its description is stated anywhere.  I assume it was deionised water – but say so. If tap water was used, give its chemical analysis as a lot of Belgian tap water is close to calcite saturation, which would perturb the interpretation enormously.

In terms of the processes that take place in weathering experiments of this type, a clear description of some of these is given in the paragraph beginning ‘Lasso…’.  Here the ability of Lasso to address linear relationships is described in the context of ecological processes, stating that biogeochemical processes are non-linear. It is a point of discussion that should be developed.  In the absence of biological factors, weathering reactions are driven by thermodynamics, with a reduction in the Gibbs free energy of the system.  These reactions are highly linear, with appropriate transformation of the data.  Curtis (1976) provides an elegant approach to this: Refer 2.   The kinetics of the reactions then is well developed by Oelkers and coauthors, exemplified by recent publications in Heřmanská et al (2022, Refer 3; 2023, Refer 4).  The bottom line is that abiological weathering reactions are not random. They are controlled by thermodynamic and kinetic processes that can be described precisely using an algebraic approach.  It would be fantastic if AI could extract evidence for this from experiments of the type reported here – you are probably the best placed research team to be able to do this.

 Other points include:

1. ‘However, care must always be taken to ensure that the minerals used do not pollute the soil with heavy metals (Dupla et al., 2023; Vienne et al., 2022).’  I agree with this sentence, but great care needs to be taken when talking about possible contamination. First, very large areas of agricultural land have basaltic rocks as the soil parent material, providing populations of millions with crops grown on effectively 100% basalt (from the inorganic point of view). Secondly, soils naturally contain ‘pollutants’ so any risk assessment must include determination of the natural background levels of these elements.    You could also cite Gastmans et al’s elegant description refer 5 ) of the geochemistry of basalt aquifers that supply millions of people in São Paulo state with clean safe drinking water.  Please consider revising the text at this point, bearing in mind the reaction you want from the reader – which should be that ‘I don’t really need to worry about this for these rocks’.
2. ‘Notwithstanding that DIC resembles TA in most organic-poor systems, in organic-rich systems, TA has an inorganic and organic component (Song et al., 2020).’  I think this could be developed, and that you should provide evidence of the amounts of DOC in your water samples. Was this information available from the FormacsHT TOC analyser?
3. Data Preprocessing section: Table A2 does not describe minerals, as implied by the text, but the rocks. Make sure you acknowledge that the individual particles of both rock and slag whose characteristics are given in Table A2 are each a composite mixture of different mineral grains.  This comes through in Table 1, where the term ‘mineral’ should be replaced by ‘rock’ or ‘material’.
4. ‘In contrast to dunite and basalt, adding steel slag reduced ∆DIC (Figure 2a).’  Did you check for the presence of carbonate?  It is easy to do, using thermal analysis, which is very sensitive (much more so than XRD).
5. ‘Steel slag typically contains free CaO and MgO (Wang et al., 2024)’.  Please check that this is not a misunderstanding of the conventional way in which chemical compositions of slags are reported – as % oxides.  It is extremely unlikely that the slag used in these experiments contained free CaO and MgO, as these oxides will hydrate or carbonate very rapidly during preparation of the material prior to use in the experiments.
6. ‘Adding more lava to the mineral mix had no clear effect..’ I don’t know what you are referring to here – please be more precise.
7. ‘Biochar was not amended’ – replace ‘amended’ with ‘added’.  ‘Amended’ suggests that you amended the biochar before use.
8. ‘Biota and biotic products.’  How dd you stop the earthworms from migrating from one pot to another or escaping altogether?  I have experience of them behaving in this way, a real nuisance.
9. ‘Second, the rock particles are grinded in the earthworms’ gizzards,’  Use ‘ground’, not ‘grinded’.

Is the study design appropriate and does the work have academic merit?

Yes

Is the work clearly and accurately presented and does it cite the current literature?

Yes

If applicable, is the statistical analysis and its interpretation appropriate?

Partly

Are all the source data underlying the results available to ensure full reproducibility?

No

Are the conclusions drawn adequately supported by the results?

Partly

Are sufficient details of methods and analysis provided to allow replication by others?

No

Reviewer Expertise:

Soil science, mineralogy, petrology, biochar

I confirm that I have read this submission and believe that I have an appropriate level of expertise to confirm that it is of an acceptable scientific standard, however I have significant reservations, as outlined above.

## References

[1] : A Chemical Classification of Volcanic Rocks Based on the Total Alkali-Silica Diagram. *Journal of Petrology* .1986;27(3) : 10.1093/petrology/27.3.745 745-750 10.1093/petrology/27.3.745

[2] : Stability of minerals in surface weathering reactions: A general thermochemical approach. *Earth Surface Processes* .1976;1(1) : 10.1002/esp.3290010107 63-70 10.1002/esp.3290010107

[3] : A comprehensive and internally consistent mineral dissolution rate database: Part I: Primary silicate minerals and glasses. *Chemical Geology* .2022;597: 10.1016/j.chemgeo.2022.120807 10.1016/j.chemgeo.2022.120807

[4] : A comprehensive and consistent mineral dissolution rate database: Part II: Secondary silicate minerals. *Chemical Geology* .2023;636: 10.1016/j.chemgeo.2023.121632 10.1016/j.chemgeo.2023.121632

[5] : Geochemical evolution of groundwater in a basaltic aquifer based on chemical and stable isotopic data: Case study from the Northeastern portion of Serra Geral Aquifer, São Paulo state (Brazil). *Journal of Hydrology* .2016;535: 10.1016/j.jhydrol.2016.02.016 598-611 10.1016/j.jhydrol.2016.02.016

# Response

**Published:** 2025-06-23

**Authors:** Iris Janssens

**Affiliations:** Computer science, Universiteit Antwerpen Faculteit Wetenschappen, Antwerp, Flanders, Belgium

We appreciate the referee's support for our innovative approach and the good suggestions. We have assessed the constructive remarks as follows:  1-2. We agree that some information was missing here. We have added more information to the Methods section and added a new section to the Extended data (section A). Specifically, 

- We have renamed lava into basanite/tephrite and dunite into peridotite; and have added a clearer description of the rocks to the methods section.  

- The Extended data (section A2) now contains the TAS diagram of the basalt and basanite/tephrite rocks is added to the Extended data (Fig. A1), references and sources of the rocks and slags (Tab. A3), the qualitative and normative compositions (XRD) (Tab. A4-5), the XRF composition (Tab. A6-7). Additional samples are now being analyzed by XRD for modal mineral results.  

- We have added the sources of the earthworms and microbes to the methods section.  

3. The biochar source (Verora, Germany) and feedstock (natural wood) are now added to the methods, and tables with its basic properties and elemental composition are added to the Extended data (Tab. A8, A9). Its IC content is given in Tab. A11 and the DIC, DOC and TA that result from columns with sand and biochar are given in Tab. A10.   4. The type of straw (wheat straw) is now added to the table, and its elemental composition is added to the Extended Data, Tab. A9.  5. We agree that knowing the full mineralogical characterization of the sand would be interesting; however, comparing TA and DIC of the input ground water (Tab. A2 in Extended Data), and the measurements after leaching through the sand bed (Tab. A10 in Extended Data), does not show an increase in TA and DIC. Hence, if calcite and aragonite or other confounding components were present in the sand, they did not leach TA and DIC. This is also expected as the mean pH of the ground water was 7.85 (Tab. A2).  6. In the revised manuscript, we have specified that the used water was natural ground water and have added tables with its properties (Tab. A1-A2 in Extended data).  (7.) We thank the reviewer for this insightful remark. We have now included this in the introduction: “Weathering reactions, such as those promoted in enhanced weathering experiments, are driven by thermodynamics and are, in absence of biotic interactions, highly linear such that their kinetics can be modelled well (Curtis et al., 1976; Heřmanská et al., 2022; Heřmanská et al., 2023). Model outputs may be transformed to yield linear relationships or normal distributions, enabling parametric statistical approaches to elucidate results from experiments or variability in time series. However, in situ, microbes will respond to the altered environmental conditions and availability of elements in soils, among others by altering their respiration, exudation of organic acids, or taking up reaction products. These microbial responses thus elicit a suite of feedbacks that the current generation of geochemical models cannot reproduce, rendering some geochemical reactions unpredictable and highly non-linear. Under these real-world conditions where reaction kinetics cannot be reproduced by existing models, machine learning that can cope with non-linear relationships may enable the attribution of observed changes in weathering reactions to potential drivers.”   Other points: 1. We thank the reviewer for these insights and have changed the sentence to: “care must always be taken to ensure that the added minerals will not increase heavy metal availabilities beyond risk-bearing thresholds (Gastmans et al; Dupla et al., 2023; Vienne et al., 2022).”  2. DOC was indeed measured. We have added the DOC measurements of the blank columns (Table A9 in Extended data). Moreover, we have added a new section to the Extended data (C.5), with a plot of the relation of ∆DIC and ∆DOC with ∆TA (Fig. C10) and used linear regression to predict ∆TA from ∆DIC and ∆DOC (Fig. C11). Results for data with steel slag have a low accuracy (either due to the limited data size, noise, or absence of a linear relation due to rapid precipitation), while a clear linear relation is found for columns without steel slag. Here, ∆DIC dominates the prediction, but ∆DOC also contributes. Given that DOC is outside the scope of our study, we did not further elaborate on this in the manuscript.  3. We indeed wrongly used minerals and rocks interchangeably. We have now corrected this issue throughout the text and in the tables.  4. Indeed, ∆DIC is low due to rapid precipitation of carbonates, which is indicated by the high ∆SIC (indicating an increase in SIC). This is shown by the high SHAP values of steel slag in the prediction of ∆SIC (Figure 3b). This is discussed in the Rocks subsection in the Results and discussion section.  5. Thank you for this suggestion, which was correct. We reformulated the text as follows: “The steel slag used had a high calcium content (Table A6 in Extended Data (Janssens et al., 2025), XRF), a portion of which was likely present in the form of calcium hydroxide (Ca(OH)₂). This phase dissolves quickly in water, increasing solution pH by releasing hydroxide ions.”

 6. We have added a reference to Figs. C3, C4 in Extended Data.  7,9. We have corrected the words. 8. A mesh was placed on top of the columns, which is now added to the methods.
